# Exploiting antigen receptor information to quantify index switching in single-cell transcriptome sequencing experiments

**DOI:** 10.1371/journal.pone.0208484

**Published:** 2018-12-05

**Authors:** Ying Yao, Asima Zia, Łukasz Wyrożemski, Ida Lindeman, Geir Kjetil Sandve, Shuo-Wang Qiao

**Affiliations:** 1 Department of Immunology, Centre for Immune Regulation, University of Oslo, Oslo, Norway; 2 K.G. Jebsen Coeliac Disease Research Centre, University of Oslo, Oslo, Norway; 3 Department of Informatics, University of Oslo, Oslo, Norway; Monash University, Australia, AUSTRALIA

## Abstract

By offering high sequencing speed and ultra-high-throughput at a low price, Illumina next-generation sequencing platforms have been widely adopted in recent years. However, an experiment with multiplexed library could be at risk of molecular recombination, known as “index switching”, which causes a proportion of the reads to be assigned to an incorrect sample. It is reported that a new advance, exclusion amplification (ExAmp) in conjunction with the patterned flow cell technology introduced on HiSeq 3000/HiSeq 4000/HiSeq X sequencing systems, potentially suffers from a higher rate of index switching than conventional bridge amplification. We took advantage of the diverse but highly cell-specific expression of antigen receptors on immune cells to quantify index switching on single cell RNA-seq data that were sequenced on HiSeq 3000 and HiSeq 4000. By utilizing the unique antigen receptor expression, we could quantify the spread-of-signal from many different wells (n = 55 from total of three batches) due to index switching. Based on full-length T cell receptor (TCR) sequences from all samples reconstructed by TraCeR and TCR gene expression quantified by Kallisto, we found index switching in all three batches of experiments investigated. The median percentage of incorrectly detected markers was estimated to be 3.9% (interquartile range (IQR): 1.7%-7.3%). We did not detect any consistent patterns of certain indices to be more prone to switching than others, suggesting that index switching is a stochastic process. Our results confirm that index switching is a problem that affects samples run in multiplexed libraries on Illumina HiSeq 3000 and HiSeq 4000 platforms.

## Introduction

Next-generation sequencing technology has been improved greatly in terms of its data output, sequencing speed, read quality and cost. As the throughput of sequencing platforms is increasing, sample multiplexing becomes practical and widely adopted by many studies. A common way to perform multiplexing is to introduce unique indices into each adaptor during library preparation, so that multiple samples can be pooled and sequenced in parallel. After sequencing, reads can be assigned to their original samples based on the indices. However, the process comes with a risk of molecular recombination, known as “index switching”, which causes some of the reads to be assigned to an incorrect sample. In 2015, Illumina adopted a new cluster generating mechanism, known as exclusion amplification (ExAmp), as well as patterned flow cells in their HiSeq 3000/4000/X instruments. By increasing cluster density on the flow cell, these new advances facilitate higher data output, more sequencing reads and shorter running time, thereby reducing sequencing cost substantially. However, because of the increased concentration of free primers produced during library preparation, it has been suggested that this promising chemistry results in a higher level of index switching than conventional bridge amplification [1]. Index switching is essentially a molecular recombination event, which is mainly driven by the presence of free-floating adaptors. A free adaptor could potentially hybridize to remaining complementary sequence of adaptor inside of a cluster and get extended. The index-switched strand can also float off and seed another nanowell on the same lane and generate a new cluster there [1]. The level of index switching depends on the purity of a library, library storage and the method of library preparation. In the Illumina white paper, the level of index switching is shown to increase in a linear fashion with the concentration of free adaptors or primers. PCR-free libraries show higher switching rates compared to library preparation that includes PCR [2].

Index switching may compromise applications that rely on accurate profiling of gene expression, especially those with the need to profile lowly expressed genes. For example, in differential expression analysis of RNA-seq data, it is usually considered to be a good practice to place samples randomly over a plate in order to minimize any potential confounding, such as the edge effect, a common phenomenon in immunoassays. To balance samples from the test group and the control group, sequencing pooled samples from each group on one lane is also preferred for the same reason. However, in an experiment impacted by index switching, those practices make it possible that reads originally from a sample in a test group might be incorrectly assigned to another sample in a control group, which weakens and distorts any real signal. In a single-cell RNA-seq experiment that mainly focuses on detecting heterogeneity among single cells, it is commonly required that hundreds or even thousands of cells are analyzed, and every one of them needs a unique index to be sequenced in a single run. Double indexing, introduced by Kircher et al. [3], increases the scope of indices by using a combination of indices for both forward and reverse adaptors. In an experiment with few samples, both indices can be unique for each sample. Reads assigned to illegitimate combinations of indices are considered to be a result of index switching, and can be easily excluded before downstream analysis. However, even the enlarged index scope with double indexing can hardly satisfy the number of cells to be sequenced in a single cell experiment. When all the combinations of indices are exhaustively used to allow more cells to be sequenced in a single run, there is no way to distinguish the incorrectly assigned reads.

To allow immune cells to be able to recognize a wide range of antigens, each naïve B and T cell expresses a unique immunoglobulin receptor (consisting of heavy and light chain) or T cell receptor (consisting of α and β chain), respectively, through somatic recombination. For human immunoglobulin heavy chain region, there are 123–129 Variable (V) gene segments [4], 27 Diversity (D) gene segments and six Joining (J) gene segments [5]. During B-cell development in the bone marrow, a stochastic process results in a recombined VDJ gene that uses one gene segment of each type. In addition to the combinatorial diversity, there is random deletion of nucleotides as well as insertion of non-germlined encoded P or N nucleotides in the junctions thus creating further diversity. A similar gene rearrangement takes place for the light chain, except for the absence of the D gene segments. Overall, the total diversity of the immunoglobulin receptor in humans is 5 x 10^13^. Likewise, T cells deploy a similar strategy for creating receptor diversity and an estimated 10^18^ different T-cell receptors can exist [6].

By using unique pairs of i5 and i7 index adaptors, the rate of index switching was estimated to be up to 2% for sequencing libraries that run on patterned flow cells using ExAmp cluster generation (2). Van der Valk et al. [7] found an average index switching rate of only 0.47% by using inline barcode ligated to reads. Sinha et al. [1] discovered that 5–10% of total mapped reads were assigned to incorrect samples in their single cell RNA-seq study using Smart-seq2 [8] for library preparation and HiSeq 4000 for sequencing. Aside from those inconsistent measurements, it remains unclear whether certain indices are more prone for switching than others. The use of unique immune receptors in immune cells provides us enough diversity for inspecting index switching in single cell RNA-seq data. From three single-cell transcriptomics libraries of human T and plasma cells (terminally differentiated B cells that express B-cell receptor) that were sequenced on the HiSeq 3000 and HiSeq 4000 platforms, we looked for index switching in the expression pattern of immune receptors. Since the receptor might be considered as a cell-specific marker, we have substantial data to quantify the impact of index switching in a single cell experimental setting.

## Method

### Ethics statement

Study participants provided written informed consent. The study was approved by Regional Committee for Medical and Health Research Ethics South-East Norway (2010/2720).

### Datasets

The data used for this study was originally generated to profile the transcriptomics of disease-specific immune cells from intestinal biopsies or peripheral blood from celiac disease patients. We used the Smart-seq2 protocol (8) to perform single-cell RNA-sequencing on 465 immune cells. The immune cells analysed included 215 HLA-DQ2: gluten-(DQ2.5-glia-α1, -α2, -ω1, and -ω2) tetramer-sorted T cells, 247 transglutaminase 2 (TG2)-specific plasma cells, and three unassigned cells in three batches. Correspondingly, Batch 1 contained one plate of 96 wells; Batch 2 contained two (Plate 2 and Plate 3); while Batch 3 contained two plates (Plate 4 and Plate 5). Except Plate 2 which only contained T cells from peripheral blood, T cells and plasma cells were randomly placed into 96-well plates as single cells by index sorting on a FACS Aria II cell sorter. The cells were retrospectively identified based on each cell’s high-dimensional immune phenotype. Double indexing was applied in library preparation, thereby every read had an index composed of two segments, indicating which row and column its source well was located. The indices were incorporated in PCR primers that were used at a final concentration of 125 nM. Free adaptors were removed by two rounds of purification with AMPure XP beads. The final sequencing library shows undetectable or negligible number of free adaptors assessed by High sensitivity DNA assay run on the BioAnalyzer (S2 Fig). Samples in Plate 1 were indexed using eight row indices and 12 column indices. After exclusive amplification (ExAmp) process, the samples were sequenced on a single lane of Illumina HiSeq 3000. In the following two batches, we used one set of indices for two plates. 12 indices were used for indexing samples on 12 columns in both Plate 2 and Plate 3, whereas 16 unique row indices were used for eight rows in Plate 2 and eight rows in Plate 3. Each plate was processed independently and the pooled indexed library from the 96 wells in each plate was combined and kept at -20C without further manipulation until sequenced on a single lane of Illumina HiSeq 4000. Likewise, Plate 4 and Plate 5 were prepared in the same way (S1 Table). The output reads were 150 bp paired-end. Average number of reads per cell was around 1.3 million.

### Quantifying expression of TR_V gene

To acquire more complete reference transcriptome for immune receptors, we downloaded human immunoglobulin (IG) and T cell receptor (TR) gene sequences in fasta format from IMGT [4], and used them to replace those in human transcriptome GRCh38. Transcript expression levels of all V gene segments were quantified by Kallisto [9]. TPM (Transcripts Per Million) values were used to represent expression for downstream analysis.

### Reconstructing T-cell and B-cell receptor

We applied TraCeR [10] to reconstruct full-length T cell receptor sequences from all samples. TraCeR is capable of extracting TCR-derived reads and mapping them to a custom-made reference database that contains all the possible combinations of V and J segments. Instead of retaining at most two TCR sequences with the largest expression per TCR locus in each single cell, which is the default setting based on a reasonable assumption in a single-cell scenario, all reconstructed TCR sequences and their corresponding expressions were used in downstream analysis to capture index switching artifacts. Similarly, we used BraCeR [11] to reconstruct full-length B cell receptor (BCR) sequences form all the samples.

### Quantifying spread of signal within and outside of the cross pattern

By using a double indexing strategy, all reads contain two indices that denote respectively a specific column and row. During demultiplexing, a read with a switched index would be incorrectly assigned to a well corresponding to the switched index. Since all index combinations were used exhaustively in all three batches, an index-switched read could not be easily identified. However, the marker gene affected by switching of one of the indices would be detected in wells in the same row or column of the origin well, but at a low expression level. Since TCR sequences are only shared between T cells belonging to the same clonotype, we expect the large majority of cells in a batch to have a unique TCR. This can be exploited to detect spread of signal originating from a well with a given TCR sequence to other wells in the same batch. For each TCR sequences detected in more than two wells in a batch, we defined the well with the highest expression in each batch as origin well. The relative expression of all wells that expressed identical TCR sequences was defined as the ratio of expression in each well to the origin well. Wells that used identical column or row indices as the origin wells were defined as within the cross, and all remaining wells were defined as outside of the cross. To show that index switching was a major cause of signal spread, we counted the number of wells within the cross that seemingly expressed the same TCR sequence as the origin well, and compared it with the number of wells expressing the same TCR sequence outside the cross.

### Testing if the spread of signal is more in the source plate than the other plate in the same batch

For each TCR marker in Batch 2 and Batch 3, we divided the above calculated relative expression in wells along the column of the origin well into those in the source plate and those in the other plate, then average the relative expressions in the two groups respectively. A paired sample t-test was conducted for testing if the estimated level of signal spreading in the source plate was higher than that in the other plate in the same batch, which was sequenced on the same lane.

### Identifying source well of signal spreading caused by index switching

For each marker used for quantifying index switching, either V gene or full-length TCR sequence, we identified the source well where the cell expressing the marker was located. Based on the expression data, we identified a well as a source of a certain marker for index switching if it met all the following criteria. 1) A well was in a position where the marker was also detected in wells in the same row or the same column (center of the cross). 2) Expression level of the marker was five times higher in this well than in any other well in the same column or the same row. 3) The cell type of the cell that was placed in the well during library preparation must match the cell type of the corresponding marker. For example, a source must be T cell while using TR_V gene segment or TCR as a marker. While using a BCR as a marker, the source cell must be a plasma cell. 4) The expression level of the marker was the highest among all the corresponding TR_V alpha or beta markers expressed in this well.

### Criteria for choosing marker, V gene segment or TCR

By assuming that the expression levels for each marker are independent, a marker was eliminated in the process of quantifying the rate of index switching if it complies to any of the following three requirements. 1) A marker was detected in less than three wells. 2) A marker was detected in more than three wells outside of the cross pattern. 3) More than two sources were identified for the same marker in a batch. In cases where both TCR sequence and TR_V gene used by it were qualified, we primarily used TR_V gene segments as markers since they are more readily detected than full-length TCR sequences and most TR_V gene segments were each only expressed by one T cell in the batch. However, in cases where the same TR_V gene segment was used by different TCRs or that cells belonging to the same clone and thus expressing the same TCR were present in the same batch, the V gene segment would be observed in wells outside of the cross pattern. Therefore, we used full-length TCR sequence reconstructed by TraCeR instead of V gene segment as a marker if a V gene segment was detected in more than one well outside of the cross pattern or more than two source wells were identified for a V gene segment.

### Calculating the rate of index switching for each index used

If a marker was detected in more than three wells outside of the cross pattern, or more than two sources were identified for the same marker in a batch, it was eliminated from further study for quantifying the rate of index switching. The reasoning was that their spreading signals are more likely to be confounded with each other’s, thus compromising the accuracy of quantification of index switching arising from a given well. When two sources of the same marker were identified in one batch, expressions of the marker on presumably overlapped positions were allocated proportionally to the sources’ expression, and then the level of signal spread was estimated for the two sources separately. The relative expression values in recipient wells (ratio of expression in the well to the expression in its source well) in the crosses for every identified marker were summed up by columns and rows, and then adjusted by dividing the number of times the well was exposed as a recipient of the identified marker.

## Results

### High rate of common TCR sequences observed in wells within the same batch

We used TraCeR (9) to reconstruct full length TCR sequences from all samples, including samples of plasma cells, because as a result of index switching we found reads mapped to TCR also in wells originally containing plasma cells. All the reconstructed receptors, including both productive and unproductive ones, were included. TCR sequences should be unique for most cells, although occasionally two cells share identical TCR sequences due to in vivo clonal expansion, these cases are relatively rare. However, we observed in our data that 182 unique full-length TCR sequences were reconstructed from 288 wells and that 76 of them were detected in more than one well. Closer inspection revealed that the shared TCR expression were most noticeable in plates belonging to the same batch and sequenced on the same lane (Table 1). This lead us to suspect that an unwanted spread-of-signal has occurred.

**Table 1:** Number of common TCRs constructed by TraCeR.

	**Plate 1**	**Plate 2**	**Plate 3**	**Plate 4**	**Plate 5**
**Plate 1**	17	1	1	2	1
**Plate 2**	1	49	14	3	3
**Plate 3**	1	14	35	2	2
**Plate 4**	2	3	2	26	15
**Plate 5**	1	3	2	15	27

Number on diagonal position is number of unique TCRs detected in every plate. Others are numbers of common TCRs in two corresponding plates. Samples in Plate 2 and Plate 3 (double outline) were indexed with the same set of column indices and sequenced on the same lane. Similarly, samples in Plate 4 and Plate 5 (double outline) were indexed with the same set of column indices. TCRs observed only in the 5 positive control wells (A1) were excluded.

There could be two major causes of signal spreading, either by index switching or by contamination. Index switching is a known problem associated with libraries sequenced on the Illumina HiSeq 3000 and 4000 platforms.

### Index switching is the main cause of the signal spreading

**Fig 1:**
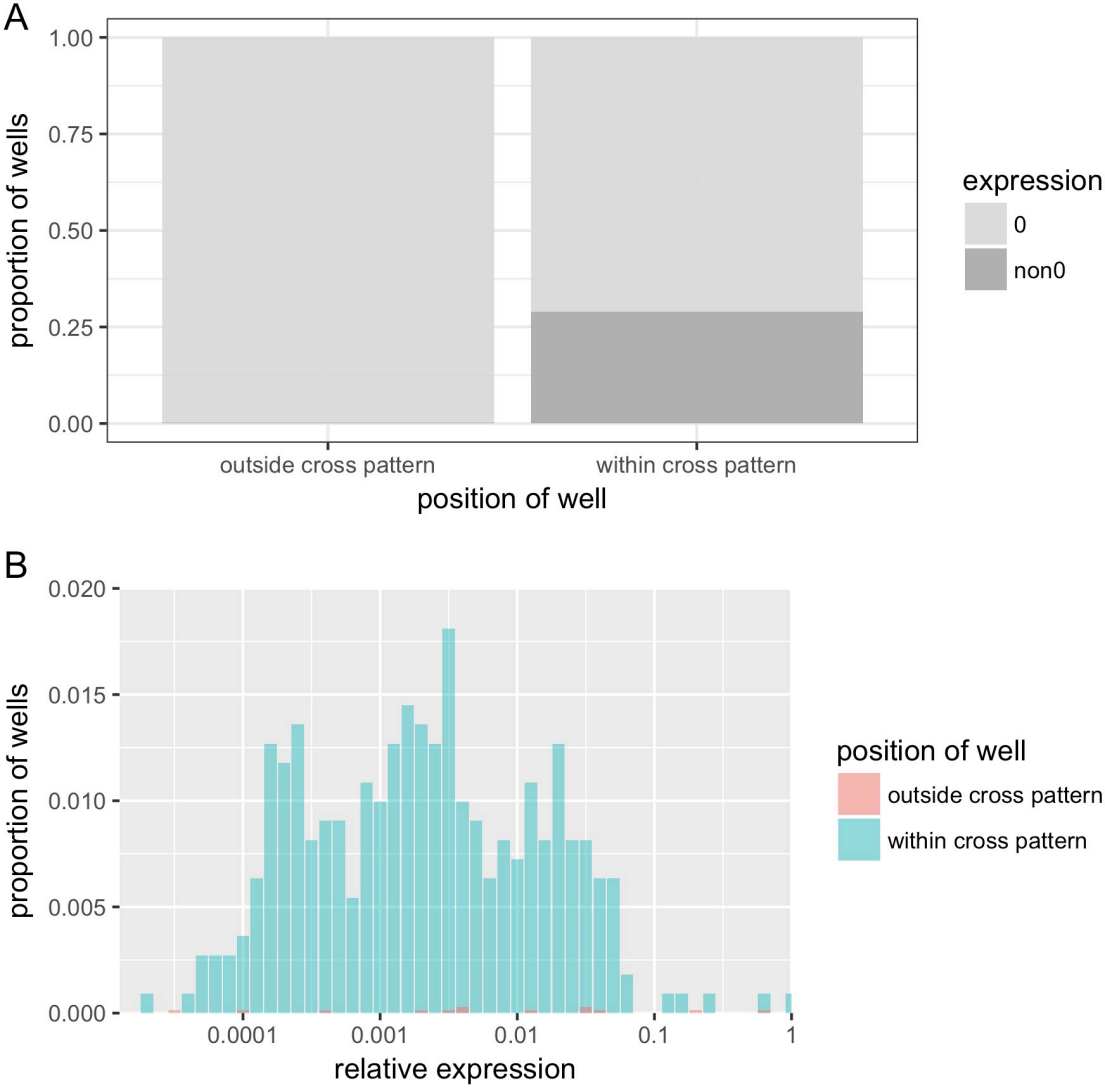
Identical TCR detected outside the origin well was found within the cross pattern. (A) Proportion of wells with detected TCR marker among wells within and outside of “cross patterns” centered at the origin wells for all TCR markers. TCR markers were detected in 320 out of 1104 (29%) within-cross wells and 13 out of 6820 (0.19%) outside-cross wells. (B). Overlaid histogram of relative expression within and outside of “cross patterns” centered at the origin wells for all TCR markers. X axis is shown on a log scale with base of 10. Wells with relative expression 0 were excluded.

Since switching of both indices is extremely rare, index switching will in most cases cause the signal to spread to wells in the same row (when column index is switched) or the same column (caused by switching of the row index). In other words, index switching will typical spread the signal from the source well to the recipient wells in a cross pattern. To verify that index switching is the main cause of the observed signal spreading, we quantified the spread of signal within the cross pattern and compared it with the signal spreading to wells outside the cross pattern. After excluding TCRs detected in less than three wells, 45 wells with the highest expression of each TCR markers were identified as origin wells. Note that these excluded TCR markers had generally very low expression—the median expression of these markers was 239 TPM (IQR 3–1531), while the median expression of the included markers was 7658 TPM (IQR 3658–14870). The same TCR sequence as in the origin wells was found in 320 out of 1104 wells (29%) in the same row or column of the origin wells, i.e. within the virtual cross patterns. In comparison, among the 6820 wells outside of the cross pattern, only 13 of them (0.19%) were found to express any of the 45 TCR markers (Fig 1A). After normalizing the counts to adjust the different number of wells within and outside a cross pattern, 99.35% of identical TCR sequences observed outside the origin wells were found within the cross patterns.

Furthermore, we excluded the origin wells and wells with 0 relative expression and then looked closely at the relative expression levels of the remaining wells. They were in most cases very low (Fig 1B). 97.7% of them were smaller than 0.05, indicating a spread-of-signal rather than true TCR duplication due to clonal expansion in vivo. Hence index switching was shown to be the main cause for identical TCRs detected in multiple wells. In addition, in a minority of wells, i.e. 7 out of 310, we observed considerable higher relative expressions ranging from 0.1 to 0.99 both within and outside of the cross patterns, indicating that there might exist in a few cases in vivo clonal expansion that resulted in identical TCR sequence in different wells.

### Contamination contributes little to the signal spreading

**Fig 2:**
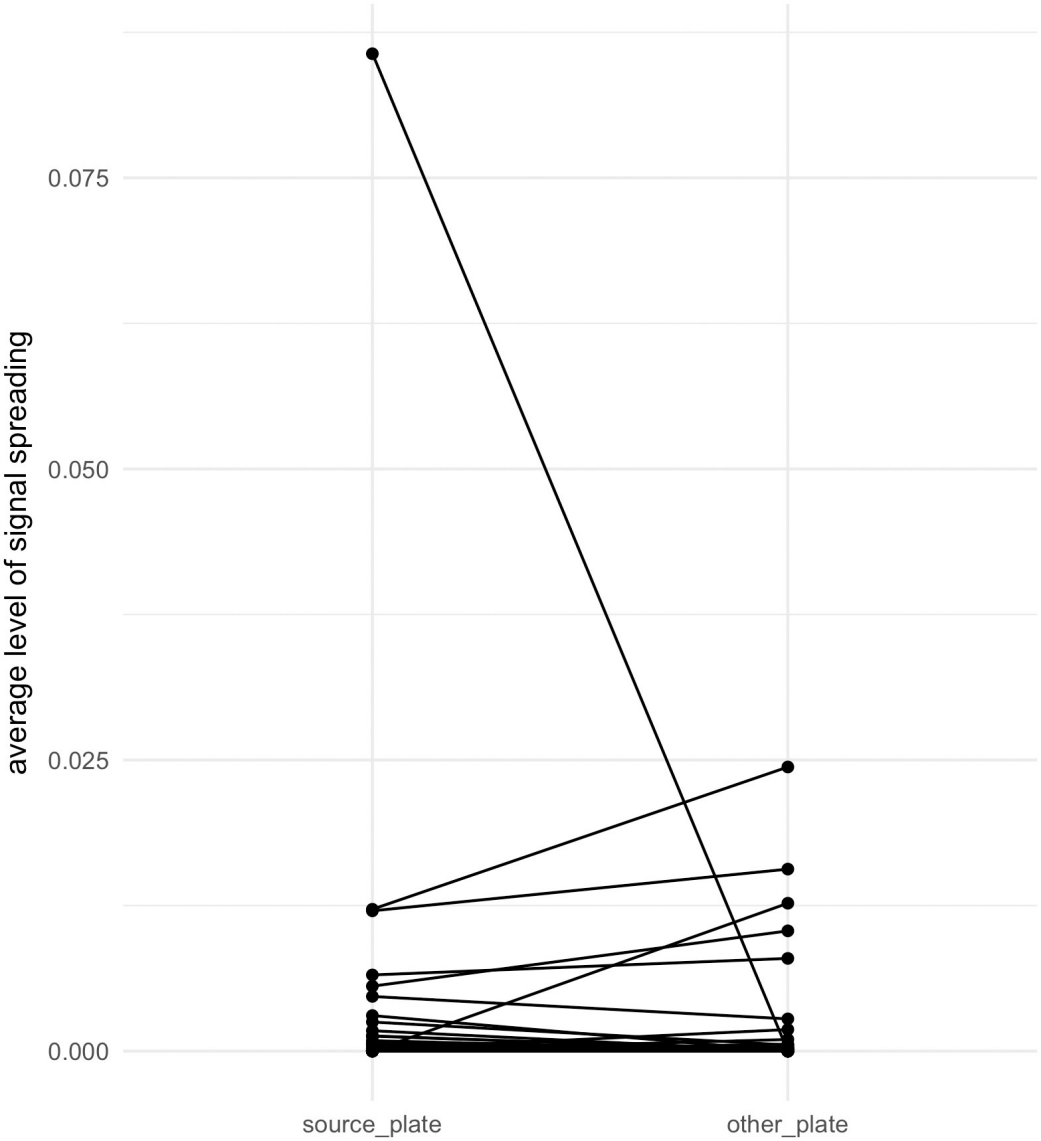
Similar average level of signal spreading to wells of the source plate compared with that to wells in the other plate in a batch sequenced on the same lane. Values on the Y axis are the average relative expression of each TCR sequence in wells in the source plate versus the average relative expression of this TCR sequence in wells in the other plate of the same batch. The wells with the highest expression of each TCR sequence (i.e. the origin wells) was excluded. P-value = 0.24 in paired samples T test.

In two of the batches, there were two 96-well plates that were each individually sorted and processed independently, including cDNA amplification, fragmentation and index barcoding by PCR. Purified libraries from each of the plates were kept frozen without further PCR rounds. The final pooled indexed libraries from each of the plates were mixed immediately before sequencing. Thus, contamination should only cause spread of signal of wells within the same plate, whereas index switching would occur both within and across the plate boundary. Hence, we assume that all spread of signal across plate boundaries is caused by index switching whereas the spread of signal within the plate is caused by index switching and possibly in addition by contamination. To test if contamination aggravated the signal spreading, we calculated the rate of TCR signal spread from the well with the highest expression in a batch to wells that shared the same column index. The spread of signal within the source plate was not significantly more than across the plates (Fig 2) by a paired samples T test (p-value = 0.24). This indicates that contamination, if any, contributes to a minor fraction of the signal spread we observe.

### Two main causes for detecting identical TCR sequences across wells

**Fig 3:**
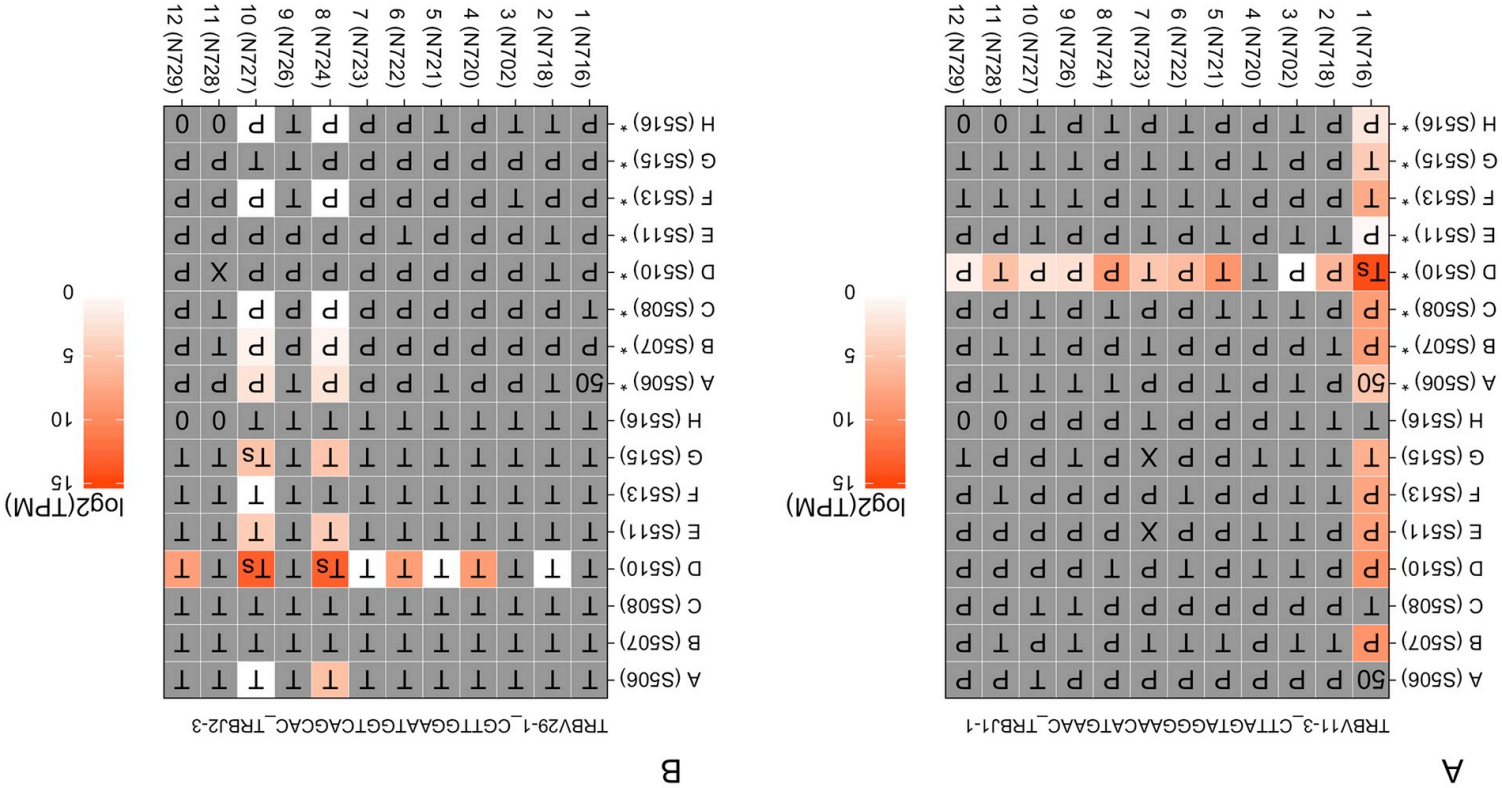
Identical TCR in multiple wells was caused by either index switching or in vivo clone expansion. (A) Expression of TraCeR-reconstructed TCR sequence TRBV11-3_CTTAGTAGGGAACATGAAC_TRBJ1-1 in Plate 4 and Plate 5 shows one unique source well. (B) Expression of TCR “TRBV29-1_CGTTGGAATGGTCAGCAC_TRBJ2-3” in Plate 2 and Plate 3 shows two independent sources of this TCR due to clone expansion in vivo. Two plates of the same batch are bound by common column indices. Upper 8 rows labeled with only letters represent Plate 2 or Plate 4; bottom 8 rows labeled with letters plus a star are from Plate 3 or Plate 5. The indices used are given in the brackets. Cell type is labeled in the corresponding well, T for T cell, P for plasma cell, 0 for empty, 50 for mixture of multiple cells and X for unknown type.

For every reconstructed TCR sequence, we profiled its expressions in all wells. Indeed, when we closely examined the expression pattern of several TCR sequences that were frequently observed in our data, we found clear cross patterns in most cases (Fig 3A). Apart from incorrectly assigned TCRs reads due to index switching, there were also a few identical TCR sequences that were truly expressed by different cells because of clonal expansion in vivo, such as cells D8 and D10 in Plate 2 (Fig 3B). In this scenario, the randomly located multiple sources give rise to spreading signals in wells along their crossing lines.

Since we have many different TCR sequences that were uniquely expressed by one or very few cells in each batch, we decided to use the immune receptor information to quantify index switching. In addition to TCR sequences, we also explore the probability for using TR_V gene segments and BCR sequences.

### TR_V gene segments are more readily detected than full-length TCR sequences

**Fig 4:**
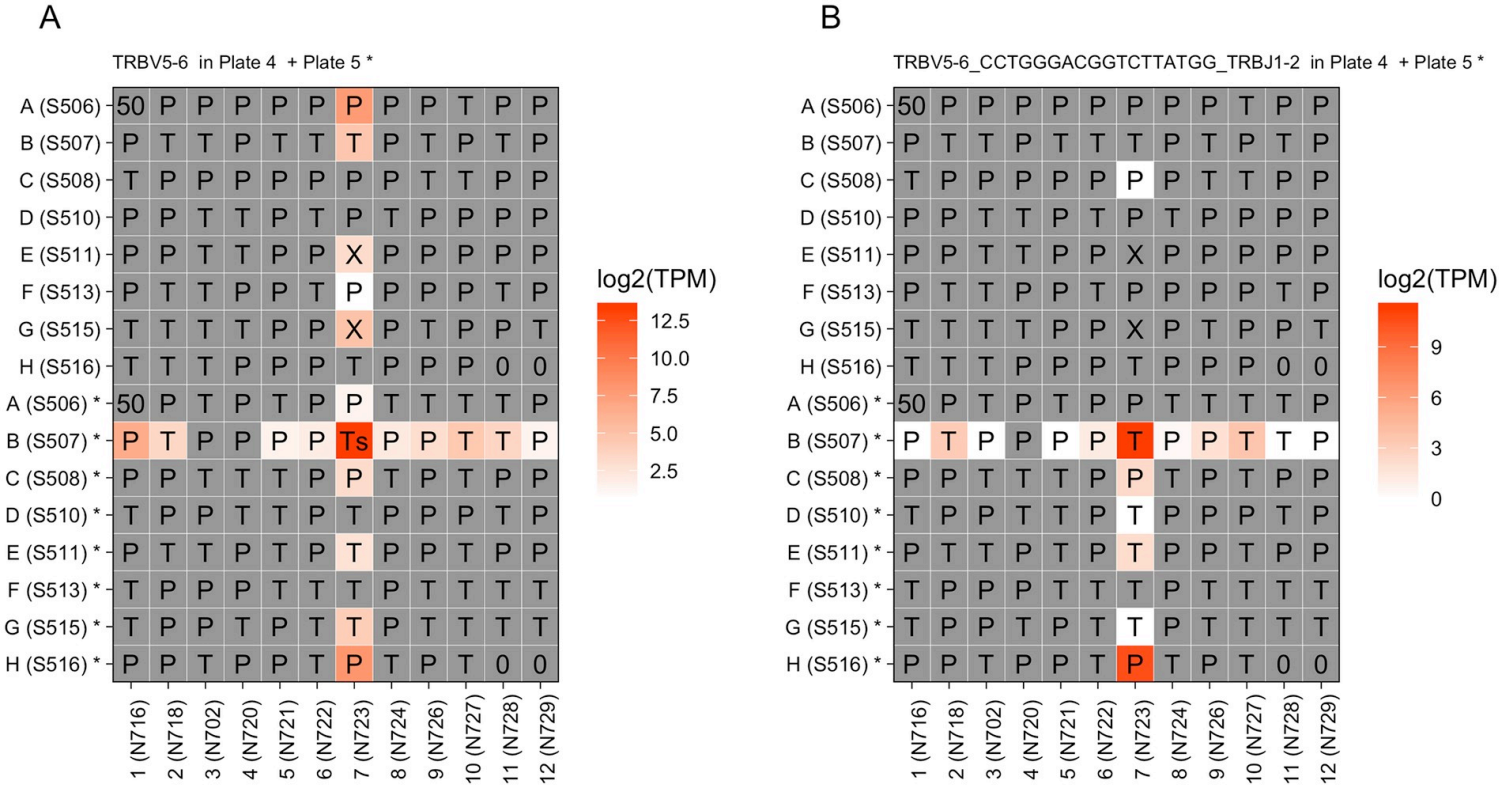
TR_V gene segments are more readily detected than full-length TCR sequences. (A). Expression of TR_V gene segment *TRBV5-6* in Plate 4 and Plate 5. (B). Expression of TCR sequence TRBV5-6_CCTGGGACGGTCTTATGG_TRBJ1-2 in Plate 4 and Plate 5. Two plates are bound by common column indices. Upper 8 rows labeled with only letters represent Plate 4; bottom 8 rows labeled with letter plus star are from Plate 5. The indices used are given in the brackets. Cell type is labeled in the corresponding well, Ts for source T cell, T for T cell, P for plasma cell, 0 for empty, 50 for mixture of multiple cells and X for unknown type.

Expression of 84 unique TR_V gene segments for TCR alpha and beta chains were detected and quantified from the 215 T cells by using Kallisto. For every single TR_V gene segment, we profiled its expression in all wells except for well A1 of every plate, where multiple cells were added as control. The results for 23 out of 84 V gene segments showed clear cross patterns in wells within the same batch (Fig 4A). The signal we can get from expression of TR_V genes is generally more complete than that from full-length TCR sequences (Fig 4B) since the TR_V genes are more readily detected.

### Reconstructed full-length antigen receptors sequences helps to separate overlapped signals, but depends on sufficient read coverage

**Fig 5:**
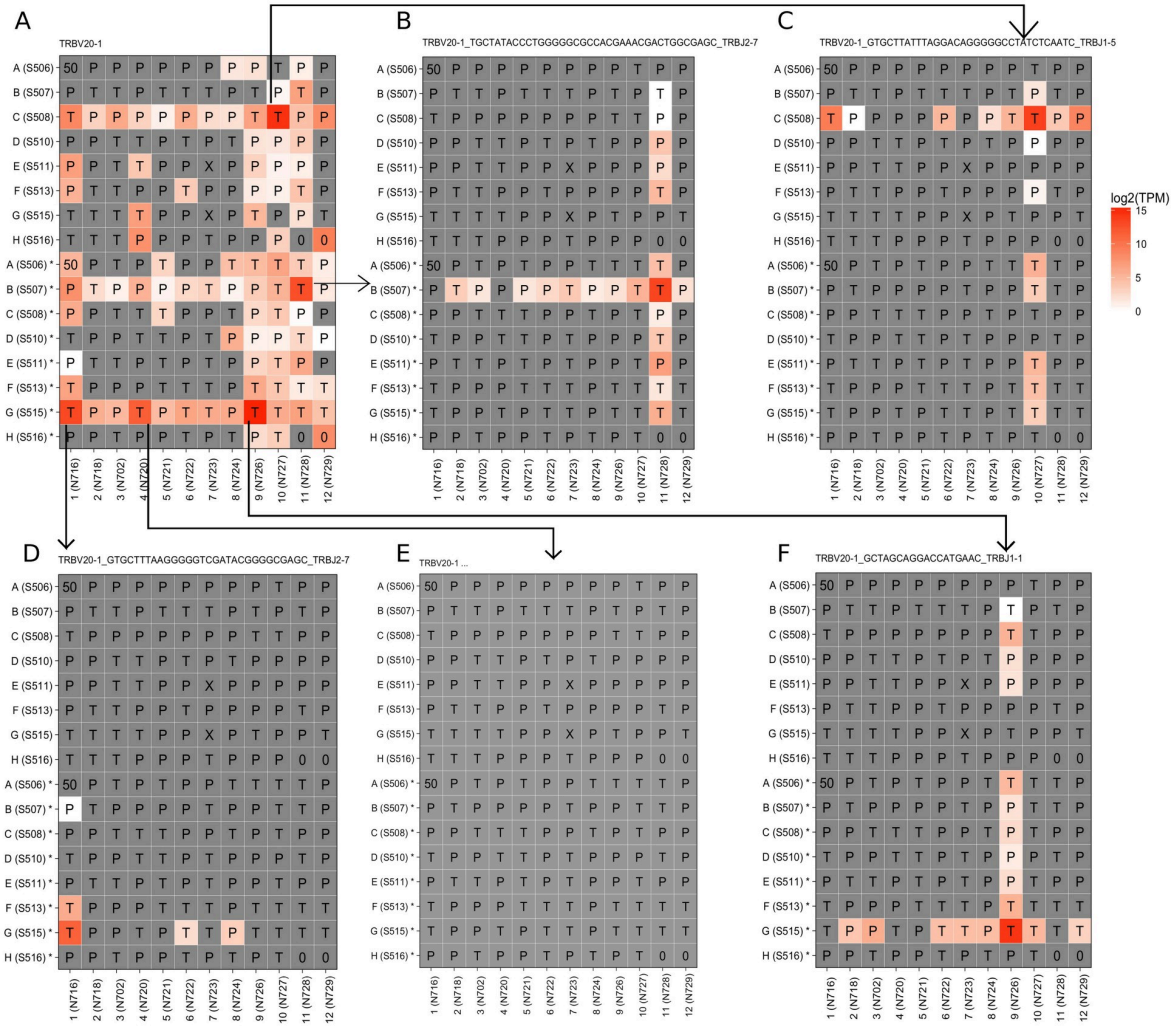
TR_V gene segment TRBV20-1 was used by multiple different TCR sequences in Plate 4 and Plate 5. (A) Expression of TR_V gene segment *TRBV20-1*. (B) Expression of TCR sequence “TRBV20-1_TGCTATACCCTGGGGGCGCCACGAAACGACTGGCGAGC_TRBJ2-7”. (C) Expression of TCR sequence “TRBV20-1_GTGCTTATTTAGGACAGGGGGCCTATCTCAATC_TRBJ1-5”. (D) Expression of TCR sequence “TRBV20-1_GTGCTATACCCTGGGGGCGCCACGAAACGACTGGCGAGC_TRBJ2-7”. (E) Potentially undetected TCR sequence that used *TRBV20-1*. (F) Expression of TCR sequence “TRBV20-1_GCTAGCAGGACCATGAAC_TRBJ1-1”.

Full-length TCR receptor sequences reconstructed by TraCeR were used to distinguish those commonly used V gene segments based on additional information such as J-chain usage and the highly diverse junctional sequence. Fig 5 shows a typical case of this. Profiling the expression of the TR_V gene segment *TRBV20-1* was too uncertain to be used for quantifying index switching. By using TraCeR, we reconstructed four unique TCR sequences that used this TR_V gene segment, and then profiled the expression of each of the four TCR sequences. Comparing with the overlapped expression while using only TR_V gene segment *TRBV20-1* (Fig 5B), the four expression plots using full-length TCR sequences (Fig 5A, 5C, 5E and 5F) were cleaner. All of them showed a clear cross pattern although one of the four TCR sequences (Fig 5A) was detected in fewer wells than the others. It was also noticeable that *TRBV20-1* was expressed at a relatively high level in well G*4 in Plate 5 with a spreading signal in wells along the same row and column. However, the expression level was not as high as in the other three sources where TCR sequences were reconstructed. It was very likely that the TCR in the well used *TRBV20-1*, and spread the signal via index switching. Due to the low number of total reads from this well, the full length TCR sequences could not be reconstructed for this particular well. To successfully reconstruct a full-length TCR sequence, TraCeR requires enough reads to cover an entire TCR chain. In a source cell with few total reads, the coverage might be insufficient to cover the entire receptor chain, in particular the part covering the non-germline encoded and recombined V(D)J which would lead to a failure to assemble a full-length TCR chain.

### B cell receptors reconstructed by BraCeR further verifies index switching

**Fig 6:**
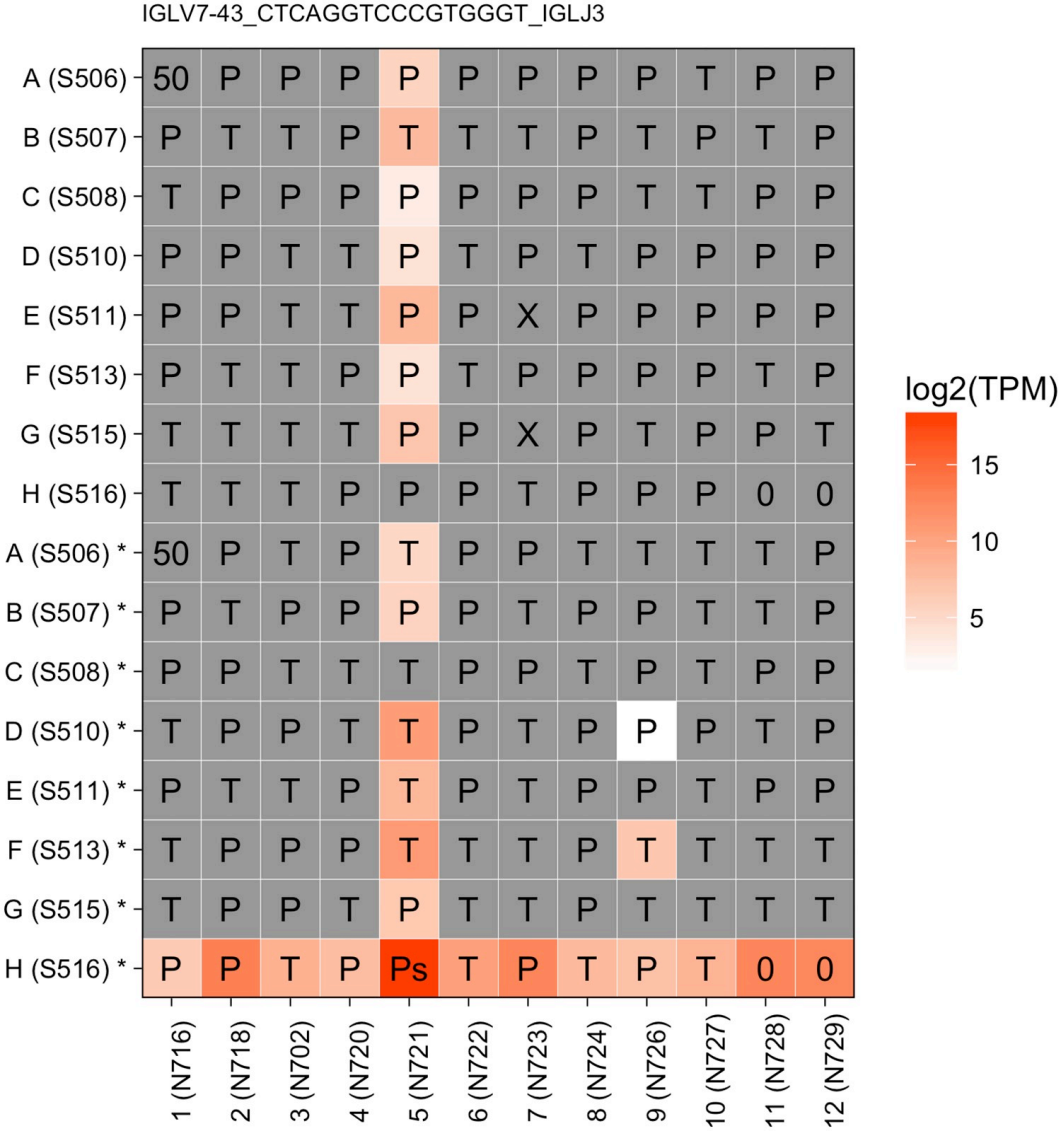
BCR reconstructed by BraCeR further verifies index switching. Expression of BraCeR-reconstructed BCR sequence “IGLV7-43_CTCAGGTCCCGTGGGT_IGLJ3” in Plate 4 and Plate 5. Two plates are bound by common column indices. Upper 8 rows labeled with only letters represent Plate 4; bottom 8 rows labeled with letter plus star are from Plate 5. The indices used are given in the brackets. Cell type is labeled in the corresponding well, T for T cell, Ps for source plasma cell, P for plasma cell, 0 for empty, 50 for mixture of multiple cells and X for unknown type.

BCR sequences expressed by plasma cells were reconstructed by BraCeR(10) and used as a marker for further verification. Since there were no plasma cells in Plate 2 of Batch 2, it was applied only to Batch 3 of our experiment. To our surprise, 521 unique BCR sequences were observed in Batch 3, in which there were only 108 plasma cells. Upon closer inspection, 322 of the BCR sequences (64%) were only detected in one cell with such a low expression that the median expression for these BCR sequences was 13.2 TPM (IQR 4.4–114.5). We speculated that aside from real cases of lowly expressed BCRs, there were considerable misassemblies by BraCeR. The high expression level of BCR genes in plasma cells combined with index switching has led to the high number of lowly expressed ‘artificial’ BCR sequences misassembled by index-switched reads from several different source wells. We therefore decided to not include any BCR markers in the quantification of index switching. However, after elimination of the BCR sequences that were detected in one well only, the results for 41 out of 199 BCR sequences showed clear cross patterns in 2 merged plates (Fig 6), confirming that indeed index switching indeed was a major problem also for BCR sequences as well.

### 3.9% of reads were affected by index switching with no preference for specific indices

**Fig 7:**
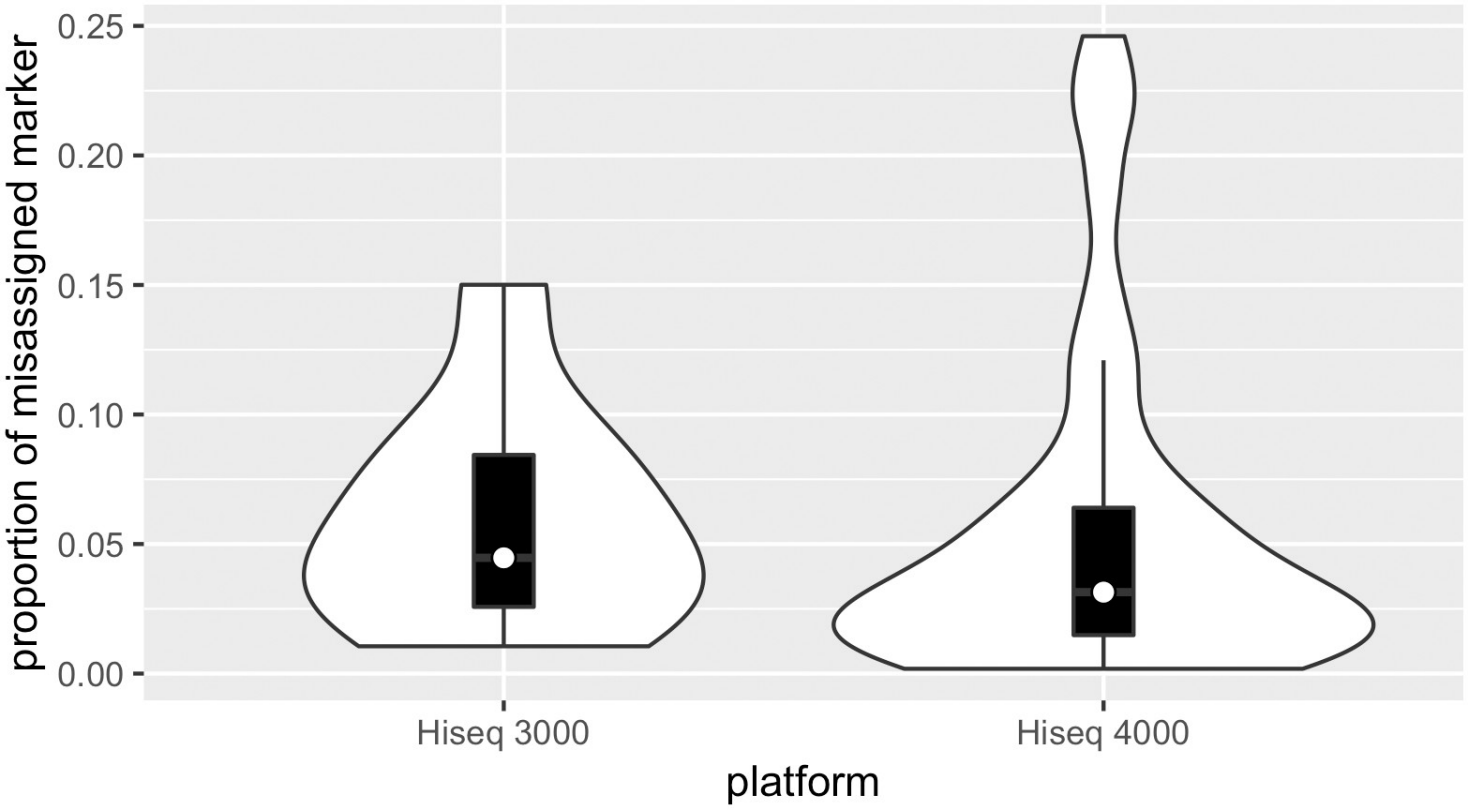
Similar signal spreading levels on Illumina HiSeq 3000 and HiSeq 4000. White spot in the center represents median value in each group. The median proportion of misassigned markers on HiSeq 3000 was 0.044. Median proportion of misassigned markers on HiSeq 4000 was 0.038.

After quality filtering, we had found in total 48 markers (15 TR_V gene and 33 TCR sequences) in 55 wells that showed unambiguous signs of spread of signal caused by index switching. This relatively large number of different markers allowed us to quantify and compare index switching between batches and between different indices. For every identified marker, we quantified the level of signal spreading by summarizing its relative expression in its recipient wells. The median percentage of incorrectly detected markers was estimated to be 3.9% (IQR: 1.7%-7.3%). For each sequencing platform, the mean level of signal spreading and systematic variation across all identified markers were assessed. We found no significant difference in signal spreading levels between Illumina HiSseq 3000 and HiSseq 4000 platforms (Fig 7), (p = 0.80, Student’s t-test).

**Fig 8:**
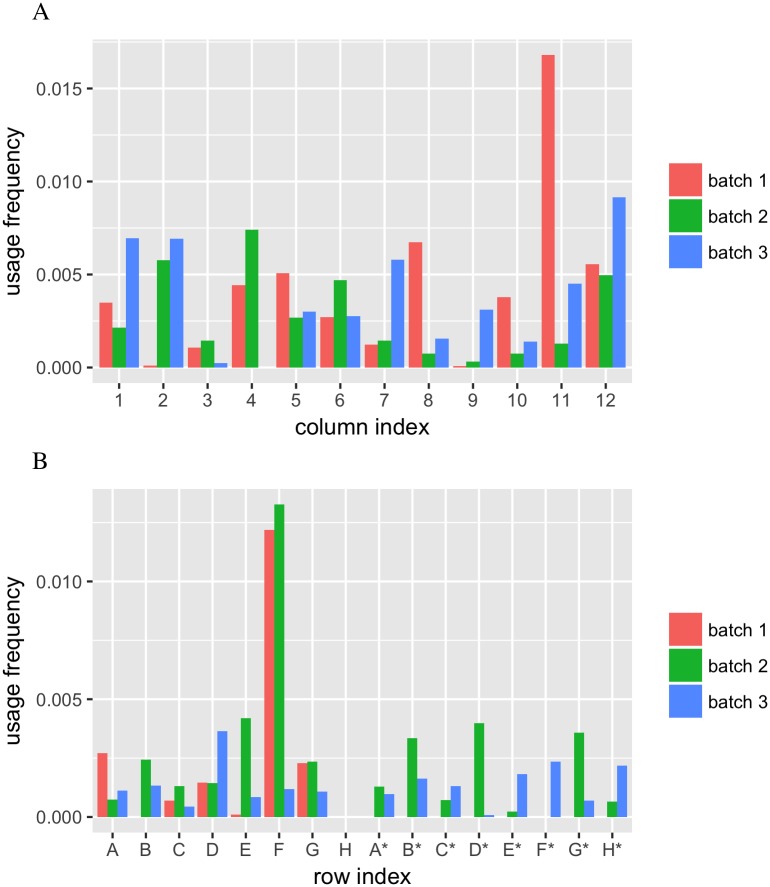
No proneness of index switching to any particular row- or column-index. (A) Estimated probability for every column-index to be used in index switching in three batches. (B) Estimated probability for every row-index to be used in index switching in three batches.

We wondered if certain indices were more prone for switching than others. To test this, for every row-index and column-index, we summarized the relative expression level of a marker for every row-index and column-index whenever the marker was detected in wells corresponding to the row-index or column-index. After subtracting those relative expressions in the source-well, we adjusted the values by dividing them by the corresponding frequencies that the row-index or column-index was not used as source. As shown in Fig 8, we did not observe any consistent proneness of index switching to any particular row- or column-index in the three batches of our experiments.

## Discussion

This study utilized the unique expression of antigen receptors by T cells and plasma cells to quantify the level of index switching. The large majority of index switching events are limited to one out of two indices and thus lead to spread-of-signal in a clear cross pattern. After confirming that there are strong cross-pattern signals in our data set, we focused the subsequent analyses on markers that allowed accurate estimation of the degree of signal spread along the cross pattern. In order to reduce noise during quantification, we used stringent criteria to select markers that appear to be clearly affected by index switching instead of other confounding factors. More specifically, by assuming that the expression levels for each marker are independent, a marker was eliminated from index switching quantification if it had any of the following three features. 1) A marker was detected in less than three recipient wells. 2) A marker was detected in more than three wells outside of the cross pattern. 3) More than two sources were identified for the same marker in a batch.

Some markers were found infrequently and in general the expression levels of these markers were very low, even in the presumptive source well with the highest expression level. This is probably due to low number of transcripts amplified in the source well during library preparation or possible misassembly during reconstruction of the TCR marker. In both cases, the information of index switching we could get by using these markers would be unreliable, as the relative expressions of the marker would be confounded either by the detection efficiency or the rate of misassembly. Therefore, we imposed the first filter to exclude those markers. However, we might introduce a slight selection bias in our estimation of index switching rates by omitting these infrequently observed markers.

There could be several causes for a marker to be detected in other wells than the source well and its cross. The most common scenario is that the marker is not specific enough, i.e. a V gene segment was used by multiple TCRs, or biological multiplication due to clonal expansion. Most of the cells that substantially expressed the same marker would be identified as sources, but we may fail to identify low-expression markers as the spread of signal could be too low to be detected. Failing to identify such a source would inflate the estimation of index switching rates since some of the signals we detected within the crosses could represent either additional unidentified sources or spread of signal from those. We therefore employed the second filter (excluding markers detected in more than three wells outside of the cross pattern) to eliminate these ‘unspecific’ markers from the quantification process. In addition, other causes such as sample contamination, dual index switching and misassembly, could also lead to signals outside the cross pattern. Though it is impossible to measure the effect of those potential causes in our experiment setting, it would be alleviated by the second filter. However, despite the second filter for marker selection, and that the result of the paired samples t-test did not indicate contamination to such an extent that it would confound the index switching effect, we cannot completely rule out the possibility of contamination, which might slightly inflate our estimated level of index switching. Similarly, unidentified additional source wells within the cross could also inflate our estimate.

Although unlikely, it is possible that index switching occurs at both ends of a read. We were unable to measure it in this study, since with such a low frequency it might not be distinguishable from those caused by sample contamination. But it would slightly deflate our estimation if there were any.

The tools that we used to reconstruct full-length antigen receptor sequence, i.e. TraCeR and BraCeR, are designed for single cell RNA-seq experiments. They are internally using Trinity [12] to assemble the receptor derived reads into a full-length receptor chain. The likelihood of misassemblies will increase as a large number of reads from different source wells are incorrectly assigned to a cell because of index switching. Compared to TCR transcripts that amounted to around 3% of all reads from T cells, a very large proportion of reads (60%-70%) from plasma cells is derived from the BCR genes due to the cell’s biological function as dedicated immunoglobulin factories. Thus, due to the extremely high expression level of BCR genes in the source wells, we observed several cases in which a BCR marker was also detected in wells outside of the cross pattern, such as D*9 and F*9 in Fig 6. However, in these cases, we were unable to ascertain whether this spread was caused by dual index switching, contamination or misassemblies.

There is a theoretical possibility that one index could convert to another as a result of PCR or sequencing errors and thus resulting in a cross pattern that is indistinguishable from that caused by index switching. However, due to the highly distinct indices that were used in our experiments, we believe it is negligible. It is especially true for this particular study, where the measurements of expressions were based on either full-length antigen receptor chains or V gene segments which are approximately twice as long as a read (150 bp). Thus, in this setting, it seems improbable that a marker would be mis-assigned due to sequencing or PCR error since it is unlikely that the exact same error would occur in a sufficient number of reads covering the marker.

It has been shown that the rate of index switching is dependent on the presence of free adaptors, usually primers with molecular size <100 bases [2]. In our three sequencing batches where indices were incorporated by PCR, the presence of low molecular-size primers was either negligible or undetectable. Compared to other published studies estimating the rate of index switching, which used a single or a handful markers, we have quantified the independent spread-of-signal of 48 cell-unique antigen receptor markers from 55 different wells. Due to the inherent variability in free adaptor amount, expression level, and how successfully signal spread is detected in the recipient wells, it is not surprising to find a relatively large variation in the estimated rate of index switch from each of these 55 wells. The median rate of 3.9% found in this study is in general agreement with the previously reported rates, ranging from 0.47% to up to 10% [1]. Thus, the index switching rate we have measured in this work is a reasonable estimate of what one could expect on the HiSeq 3000 and HiSeq 4000 platforms when libraries are prepared according to ‘best practices’.

## Conclusions

In this study, we estimated the level of read misassignment due to index switching in three single-cell transcriptomics libraries sequenced on the Illumina HiSeq 3000 or HiSeq 4000 sequencing platforms. We took advantage of the unique antigen receptor genes expressed by T cells and B cells and could thus quantify the level of signal spreading from 48 different gene markers in 55 different wells. The median percentage of incorrectly assigned markers was estimated to be 3.9% (IQR 1.7%-7.3%). There was no significant difference in index switching level between Illumina HiSeq 3000 and HiSeq 4000 sequencing platforms. Furthermore, we did not detect any consistent pattern of certain indices to be more prone for switching than others, suggesting that index switching is a stochastic process. Our study confirms that index switching is a problem that affects all samples run in multiplexed libraries on Illumina HiSeq 3000 and HiSeq 4000 platforms, and suggests that immune cell receptor information can be utilized to quantify the level of index switching for a given setup of single cell experiments.

## Supporting information

S1 Table Diagram showing the usage of indices and cell type in all wells in the 3 batches. (DOCX)

S2 Table Number of common TR_V detected. Number on diagonal position is number of unique TCRs detected in each plate. Others are numbers of common TCRs in two corresponding plates. Samples in Plate 2 and Plate 3 were indexed with the same set of column indices and sequenced on the same lane. Similarly, samples in plate 4 and plate 5 were indexed with the same set of column indices and sequenced on the same lane.
(DOCX)

S1 Fig Expression of all markers used to quantify index switching, including T cell receptor V-gene segment and TraCeR-reconstructed T cell receptor, in 3 batches. Cell type is labeled in the corresponding well, T for T cell, P for plasma cell, 0 for empty, 50 for mixture of multiple cells and X for unknown type. Ts for T cell identified as a source of signal spreading.
(PDF)

S2 Fig BioAnalyzer traces of sequencing-ready libraries show negligible amount of small-molecular primer-dimers. After Tagmentation, adaptors including indices were incorporated by PCR. The 96 samples from each plate were then pooled and free primers were removed by two rounds of purification with AMPure XP beads. The pooled libraries were quality-checked on the BioAnalyzer by the High-sensitivity DNA kit before kept frozen at -20C until sequencing.
(PDF)
