# Supplementary material for: Exploiting antigen receptor information to quantify index switching in single-cell transcriptome sequencing experiments
Source: PLoS One. 2018 Dec 5;13(12):e0208484. doi: 10.1371/journal.pone.0208484 (PMC6281226; doi:10.1371/journal.pone.0208484)
Supplement: S1 Table — (DOCX) [file pone.0208484.s001.docx]

Supplementary Table 1

| **BATCH1** |  | N716 | N718 | N702 | N720 | N721 | N722 | N723 | N724 | N726 | N727 | N728 | N729 |
| --- | --- | --- | --- | --- | --- | --- | --- | --- | --- | --- | --- | --- | --- |
|  |  | **1** | **2** | **3** | **4** | **5** | **6** | **7** | **8** | **9** | **10** | **11** | **12** |
| S506 | **A** | 50 | P | P | P | T | T | P | P | P | P | T | T |
| S507 | **B** | P | P | P | P | P | P | X | P | P | P | P | P |
| S508 | **C** | P | P | T | P | P | T | P | P | T | T | P | P |
| S510 | **D** | T | P | P | T | T | T | T | P | T | P | P | P |
| S511 | **E** | P | T | P | P | P | P | P | P | P | T | P | P |
| S513 | **F** | P | P | P | P | T | P | P | P | T | T | P | P |
| S515 | **G** | T | P | T | P | T | T | P | P | P | P | T | P |
| S516 | **H** | P | T | P | T | T | T | P | P | P | P | 0 | 0 |
|  |  |  |  |  |  |  |  |  |  |  |  |  |  |
|  |  |  |  |  |  |  |  |  |  |  |  |  |  |
| **BATCH2** |  | N716 | N718 | N702 | N720 | N721 | N722 | N723 | N724 | N726 | N727 | N728 | N729 |
|  |  | **1** | **2** | **3** | **4** | **5** | **6** | **7** | **8** | **9** | **10** | **11** | **12** |
| S506 | **A** | 50 | T | T | T | T | T | T | T | T | T | T | T |
| S507 | **B** | T | T | T | T | T | T | T | T | T | T | T | T |
| S508 | **C** | T | T | T | T | T | T | T | T | T | T | T | T |
| S510 | **D** | T | T | T | T | T | T | T | T | T | T | T | T |
| S511 | **E** | T | T | T | T | T | T | T | T | T | T | T | T |
| S513 | **F** | T | T | T | T | T | T | T | T | T | T | T | T |
| S515 | **G** | T | T | T | T | T | T | T | T | T | T | T | T |
| S516 | **H** | T | T | T | T | T | T | T | T | T | T | 0 | 0 |
| S502 | **A*** | 50 | T | P | P | T | P | P | P | T | P | P | P |
| S503 | **B*** | P | P | P | P | P | P | P | P | P | P | T | P |
| S505 | **C*** | T | P | P | P | P | P | P | P | P | P | T | P |
| S517 | **D*** | P | T | P | P | P | P | P | P | P | P | X | P |
| S518 | **E*** | P | T | P | P | P | T | P | P | P | P | P | P |
| S520 | **F*** | P | P | T | P | P | P | P | P | T | P | P | P |
| S521 | **G*** | P | P | P | P | P | P | P | P | T | T | P | P |
| S522 | **H*** | P | T | T | P | T | P | P | P | T | P | 0 | 0 |
|  |  |  |  |  |  |  |  |  |  |  |  |  |  |
|  |  |  |  |  |  |  |  |  |  |  |  |  |  |
| **BATCH3** |  | N716 | N718 | N702 | N720 | N721 | N722 | N723 | N724 | N726 | N727 | N728 | N729 |
|  |  | **1** | **2** | **3** | **4** | **5** | **6** | **7** | **8** | **9** | **10** | **11** | **12** |
| S506 | **A** | 50 | P | P | P | P | P | P | P | P | T | P | P |
| S507 | **B** | P | T | T | P | T | T | T | P | T | P | T | P |
| S508 | **C** | T | P | P | P | P | P | P | P | T | T | P | P |
| S510 | **D** | P | P | T | T | P | T | P | T | P | P | P | P |
| S511 | **E** | P | P | T | T | P | P | X | P | P | P | P | P |
| S513 | **F** | P | T | T | P | P | T | P | P | P | P | T | P |
| S515 | **G** | T | T | T | T | P | P | X | P | T | P | P | T |
| S516 | **H** | T | T | T | P | P | P | T | P | P | P | 0 | 0 |
| S502 | **A*** | 50 | P | T | P | T | P | P | T | T | T | T | P |
| S503 | **B*** | P | T | P | P | P | P | T | P | P | T | T | P |
| S505 | **C*** | P | P | T | T | T | P | P | T | P | T | P | P |
| S517 | **D*** | T | P | P | T | T | P | T | P | P | P | T | P |
| S518 | **E*** | P | T | T | P | T | P | T | P | P | T | P | P |
| S520 | **F*** | T | P | P | P | T | T | T | P | T | T | T | T |
| S521 | **G*** | T | P | P | T | P | T | T | P | T | T | T | T |
| S522 | **H*** | P | P | T | P | P | T | P | T | P | T | 0 | 0 |

**Supplementary table 1. Diagram showing the usage of indices and cell type in all wells in the 3 batches**
