# Supplementary material for: Exploiting antigen receptor information to quantify index switching in single-cell transcriptome sequencing experiments
Source: PLoS One. 2018 Dec 5;13(12):e0208484. doi: 10.1371/journal.pone.0208484 (PMC6281226; doi:10.1371/journal.pone.0208484)
Supplement: S2 Table — Number on diagonal position is number of unique TCRs detected in each plate. Others are numbers of common TCRs in two corresponding plates. Samples in Plate 2 and Plate 3 were indexed with the same set of column indices and sequenced on the same lane. Similarly, samples in plate 4 and plate 5 were indexed with the same set of column indices and sequenced on the same lane. (DOCX) [file pone.0208484.s002.docx]

- Supplementary Table 2

|  | - Plate 1 | - Plate 2 | - Plate 3 | - Plate 4 | - Plate 5 |
| --- | --- | --- | --- | --- | --- |
| - Plate 1 | - 28 | - 22 | - 18 | - 13 | - 16 |
| - Plate 2 | - 22 | - 74 | - 55 | - 31 | - 32 |
| - Plate 3 | - 18 | - 55 | - 59 | - 26 | - 25 |
| - Plate 4 | - 13 | - 31 | - 26 | - 35 | - 27 |
| - Plate 5 | - 16 | - 32 | - 25 | - 27 | - 35 |

- **Supplementary table 2. Number of common TR_V detected:** Number on diagonal position is number of unique TCRs detected in each plate. Others are numbers of common TCRs in two corresponding plates. Samples in plate 2 and plate 3 were indexed with the same set of column indices and sequenced on the same lane. Similarly, samples in plate 4 and plate 5 were indexed with the same set of column indices and sequenced on the same lane.
