# Supplementary figures and images for: Exploiting antigen receptor information to quantify index switching in single-cell transcriptome sequencing experiments
Source: PLoS One. 2018 Dec 5;13(12):e0208484. doi: 10.1371/journal.pone.0208484 (PMC6281226; doi:10.1371/journal.pone.0208484)

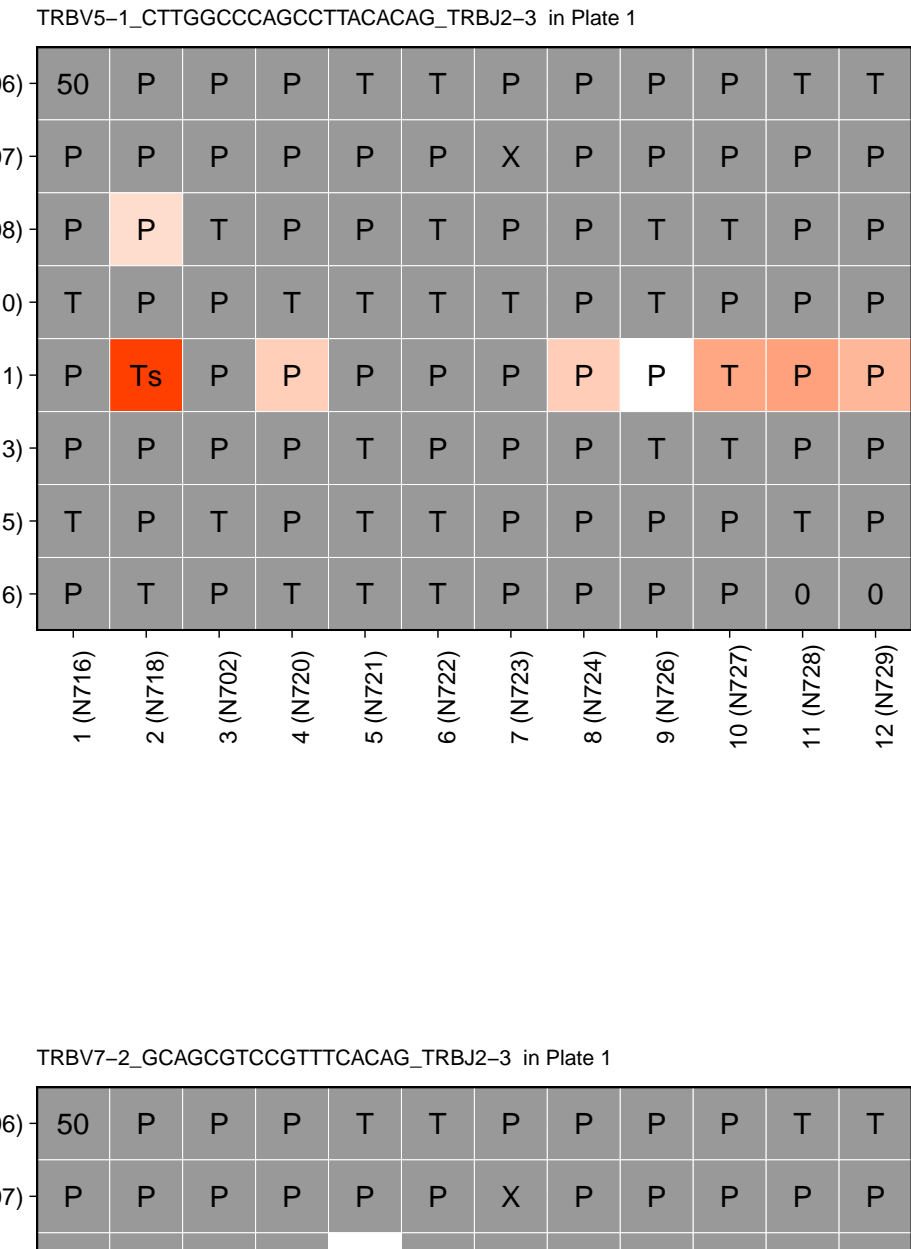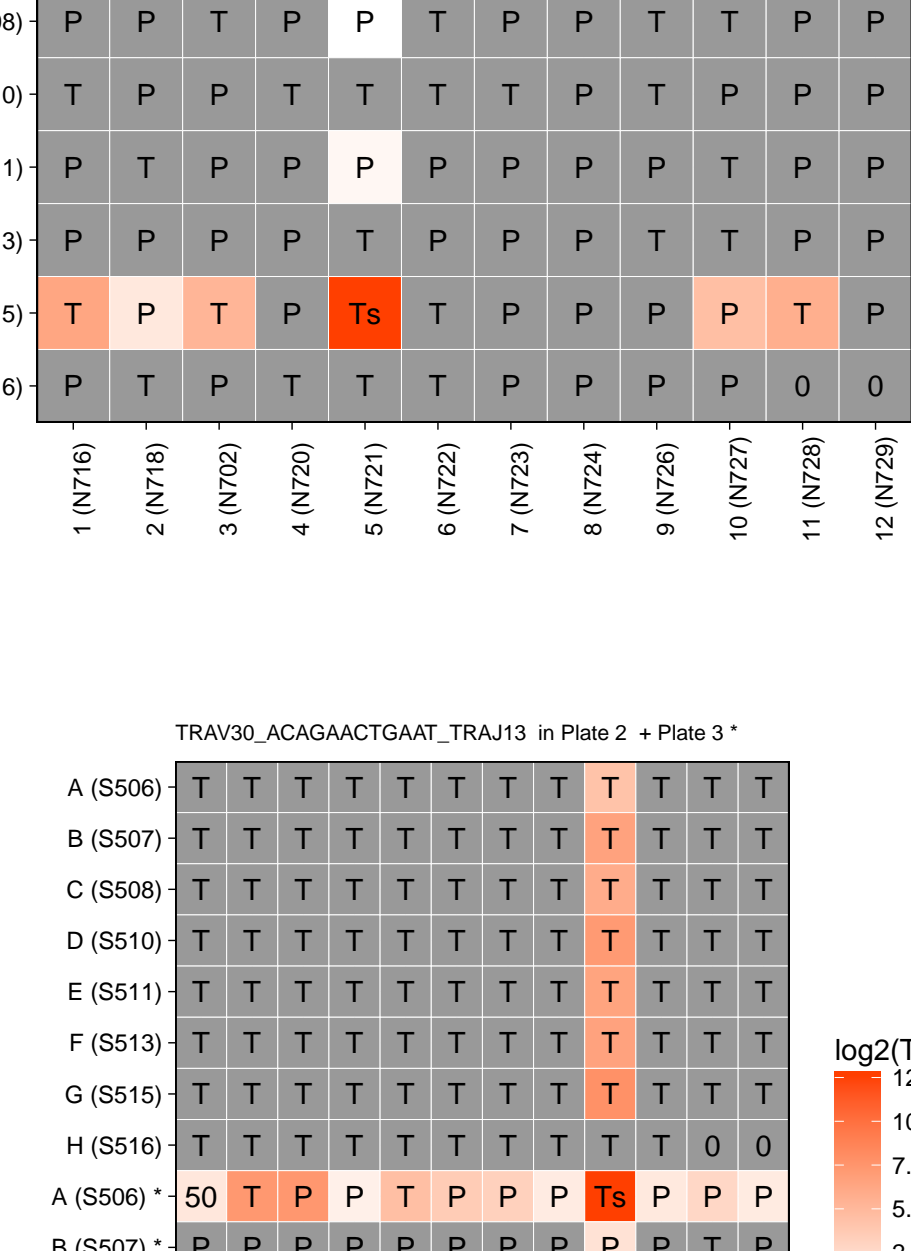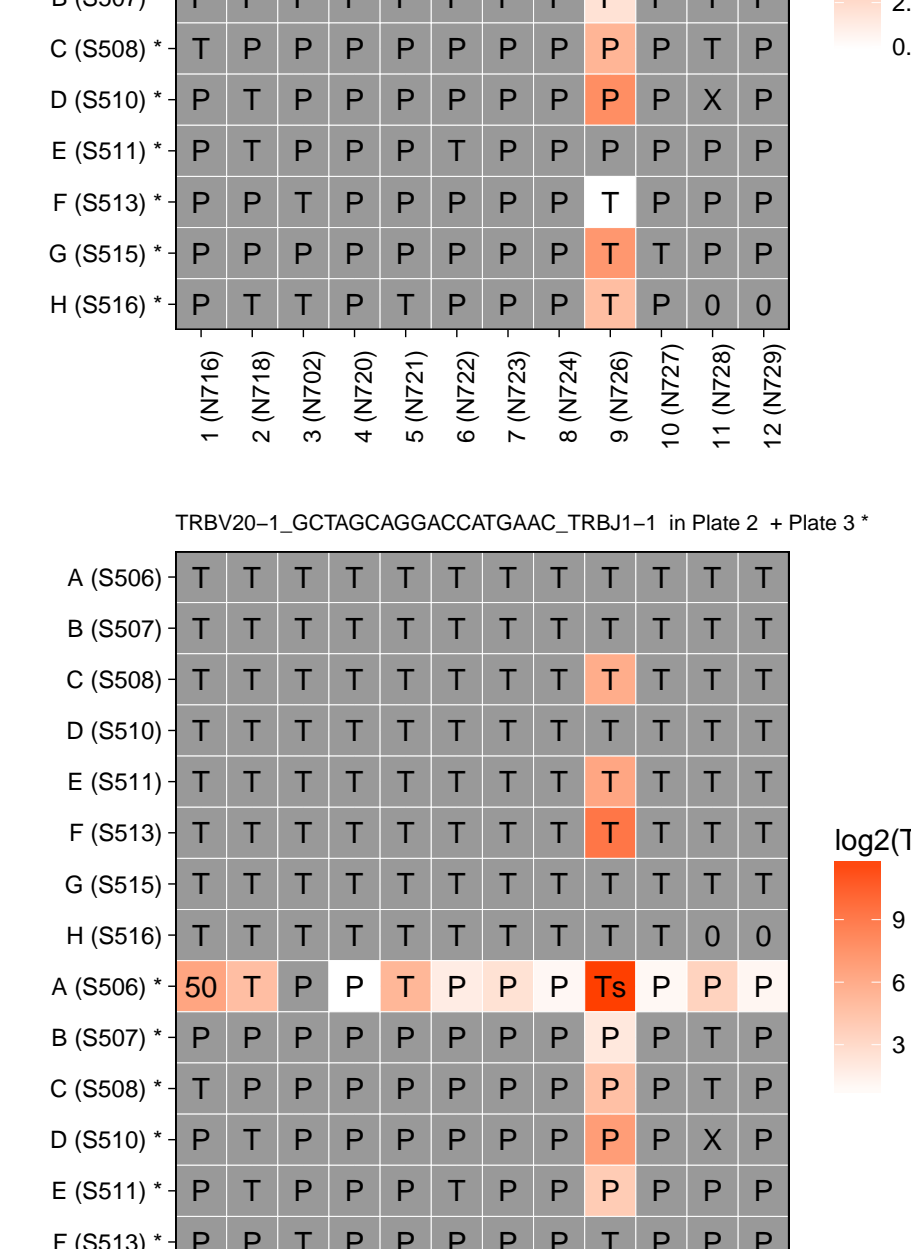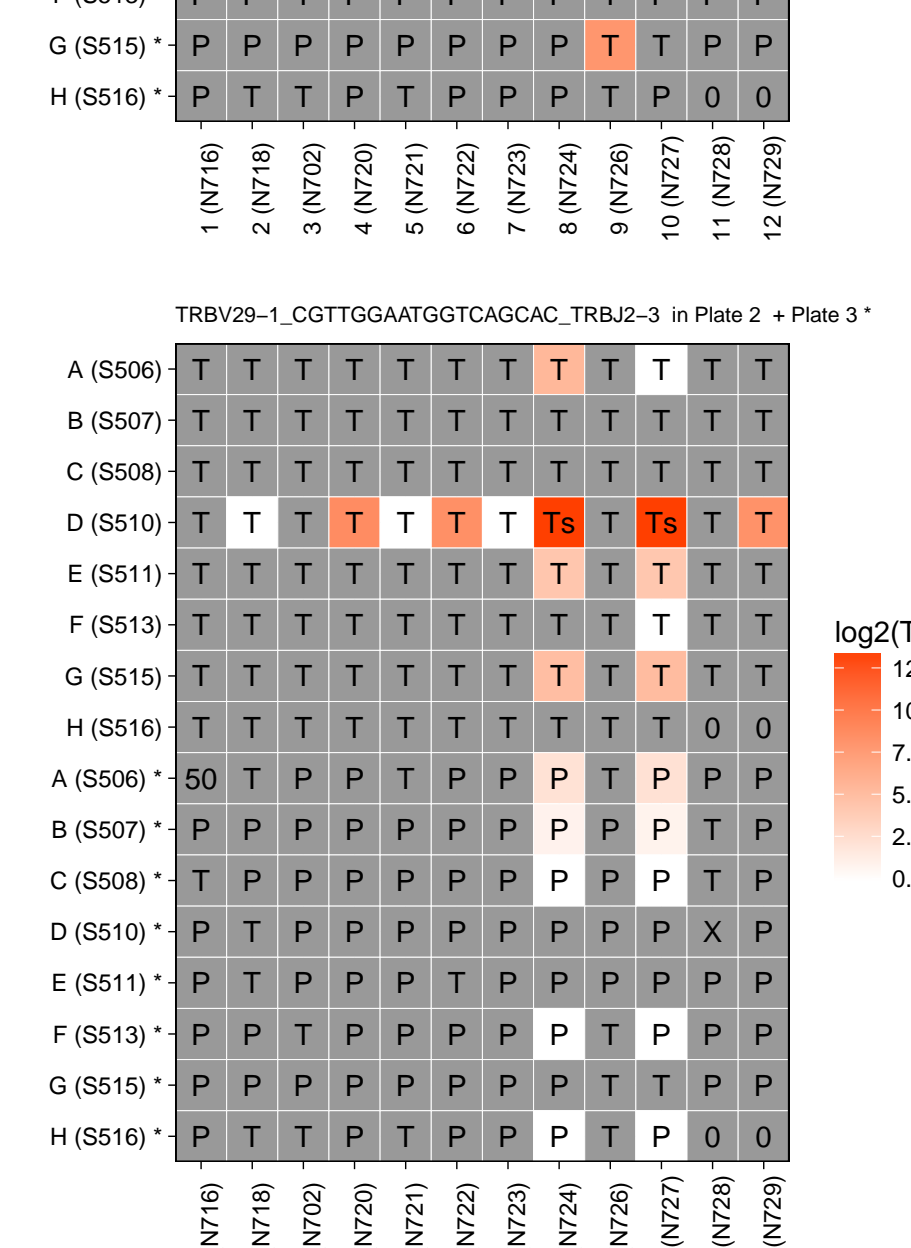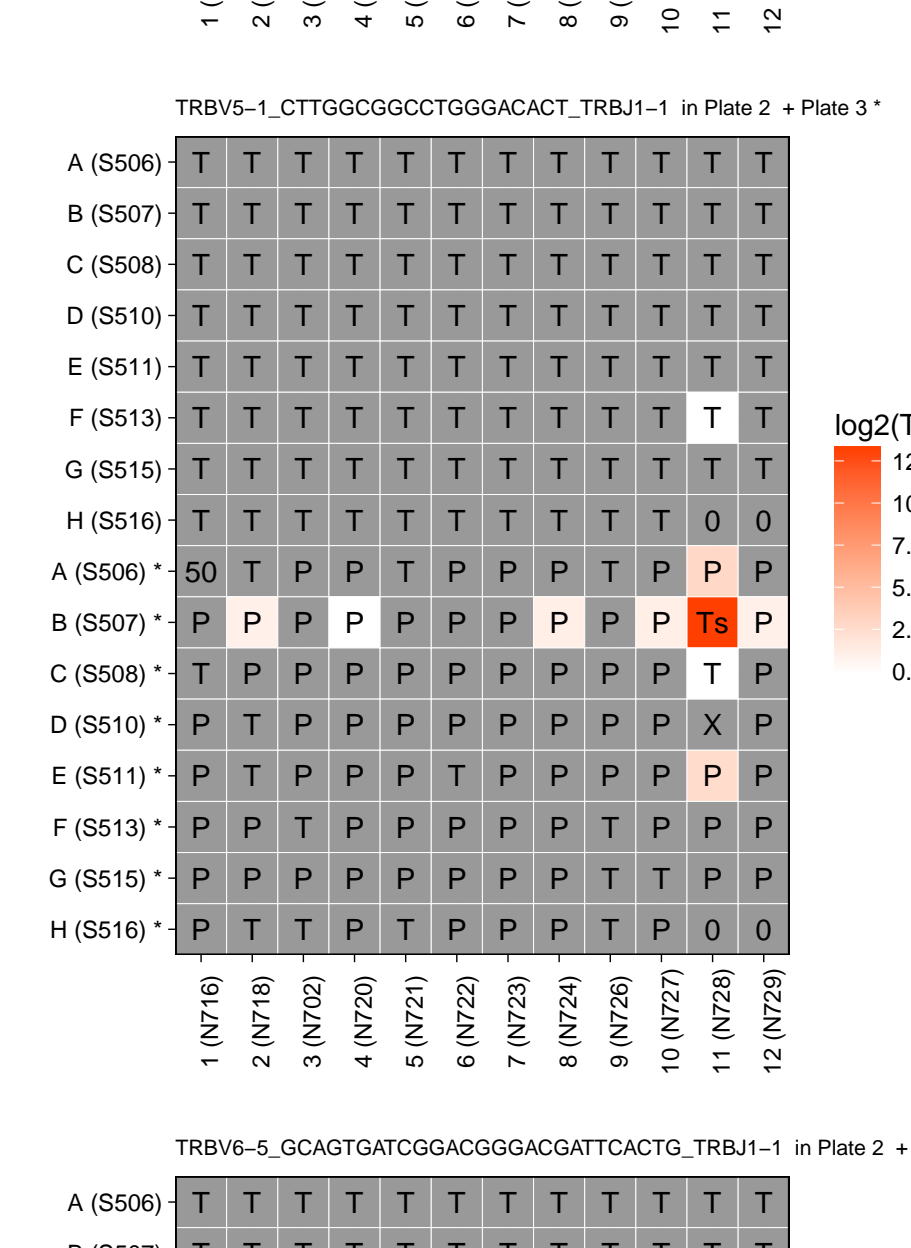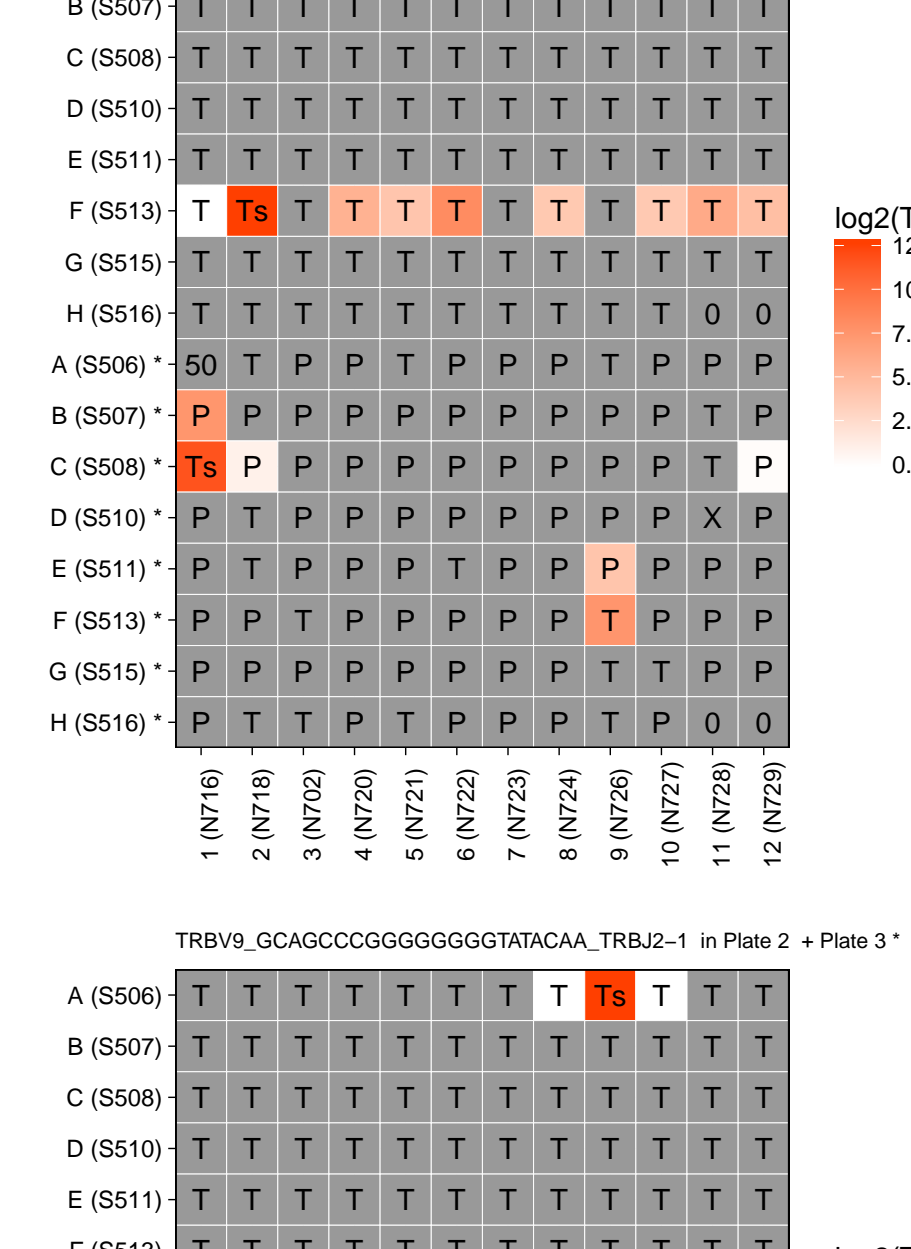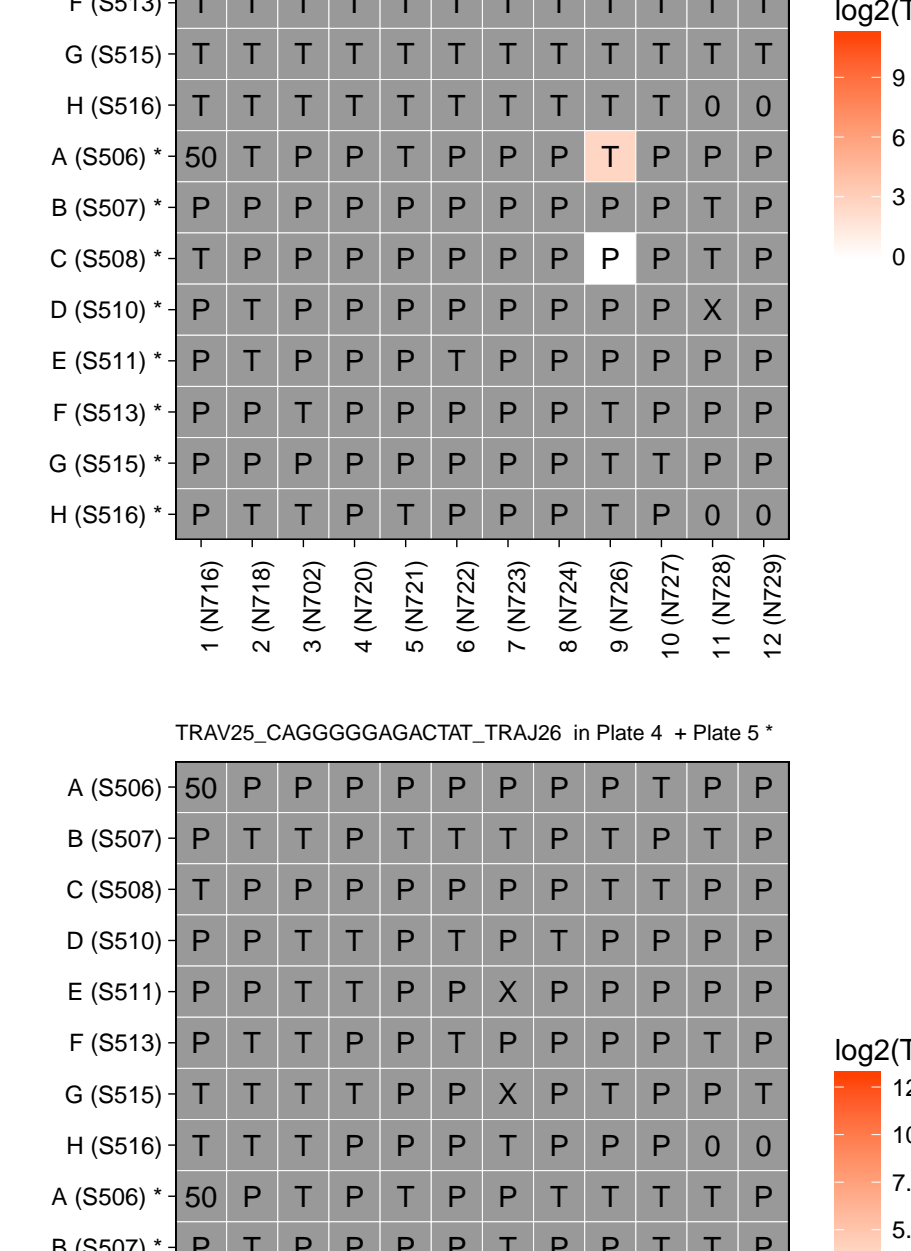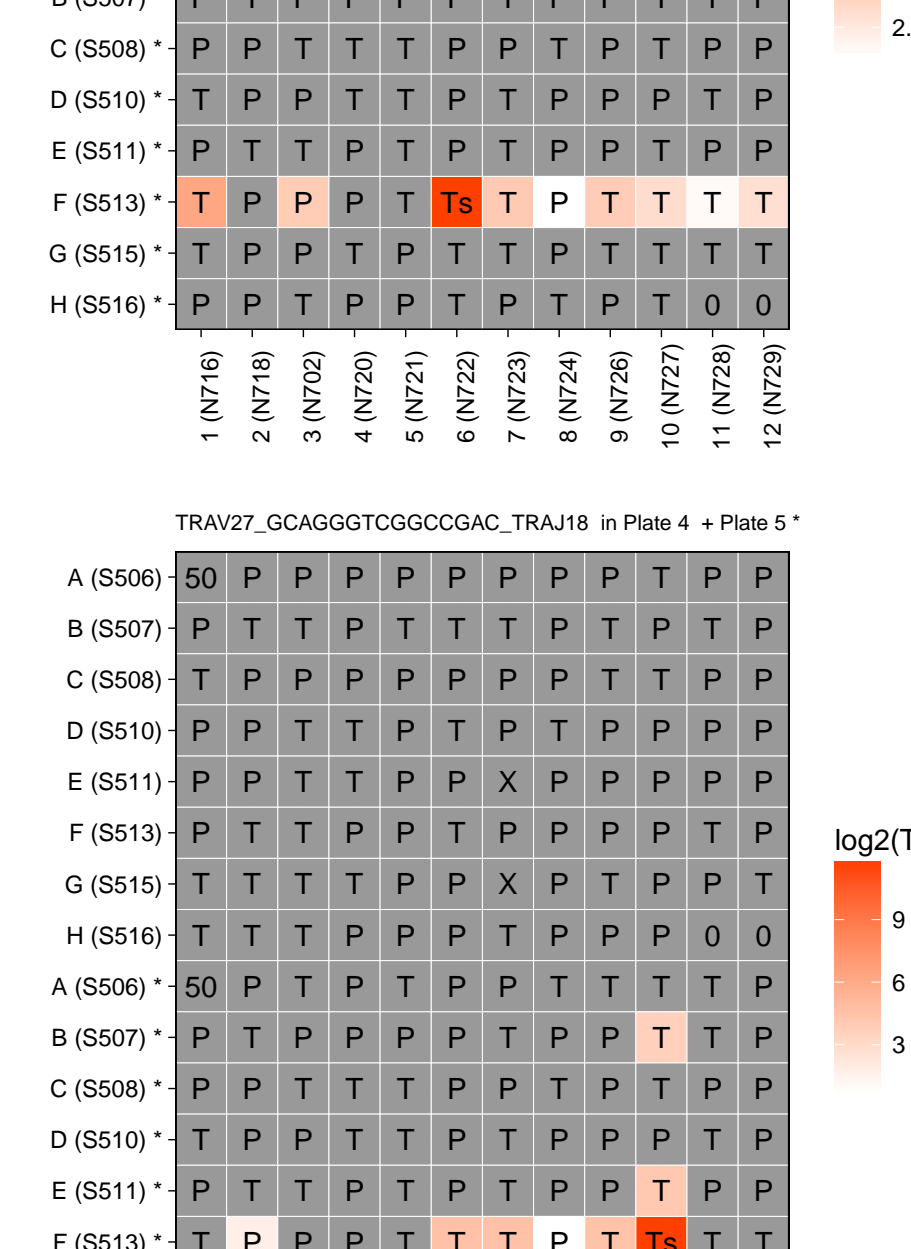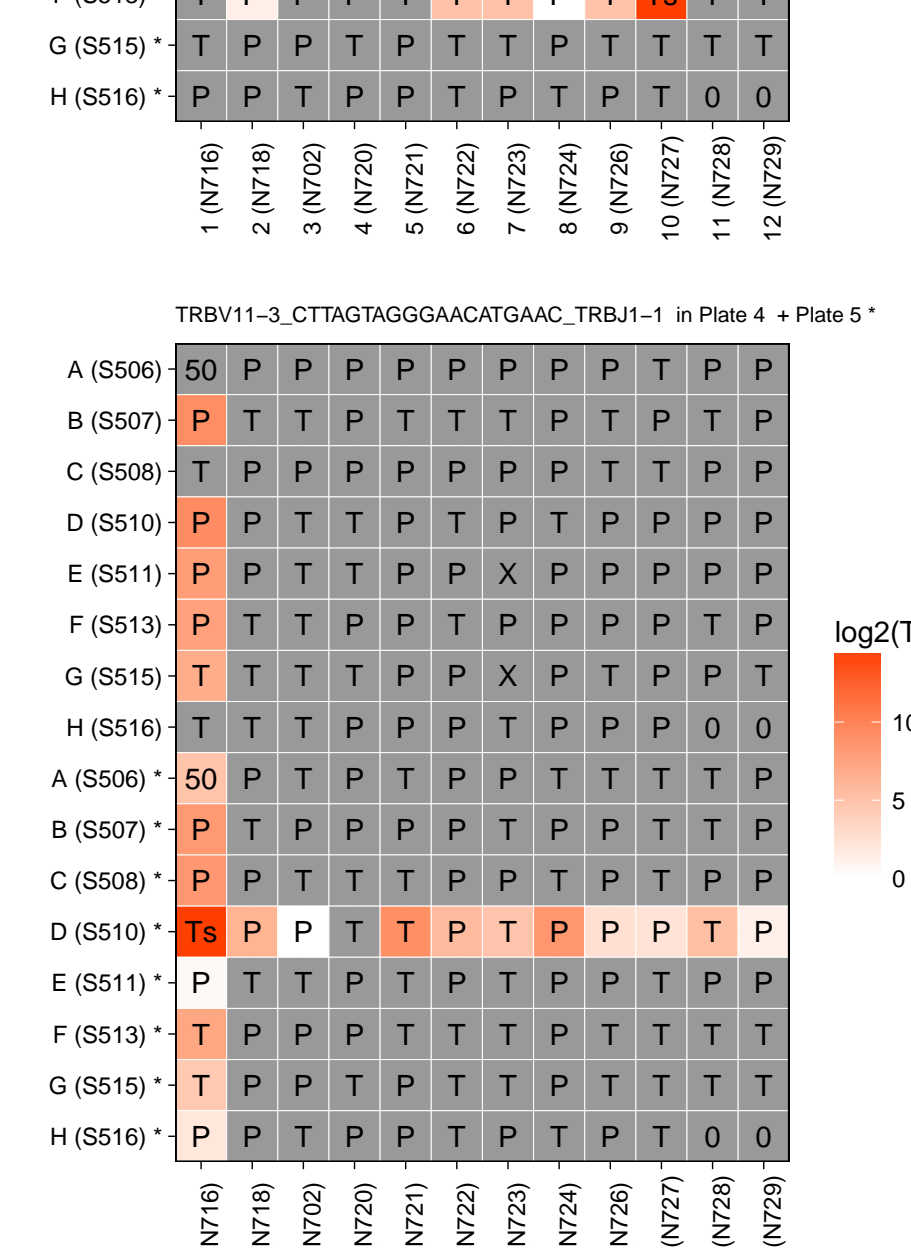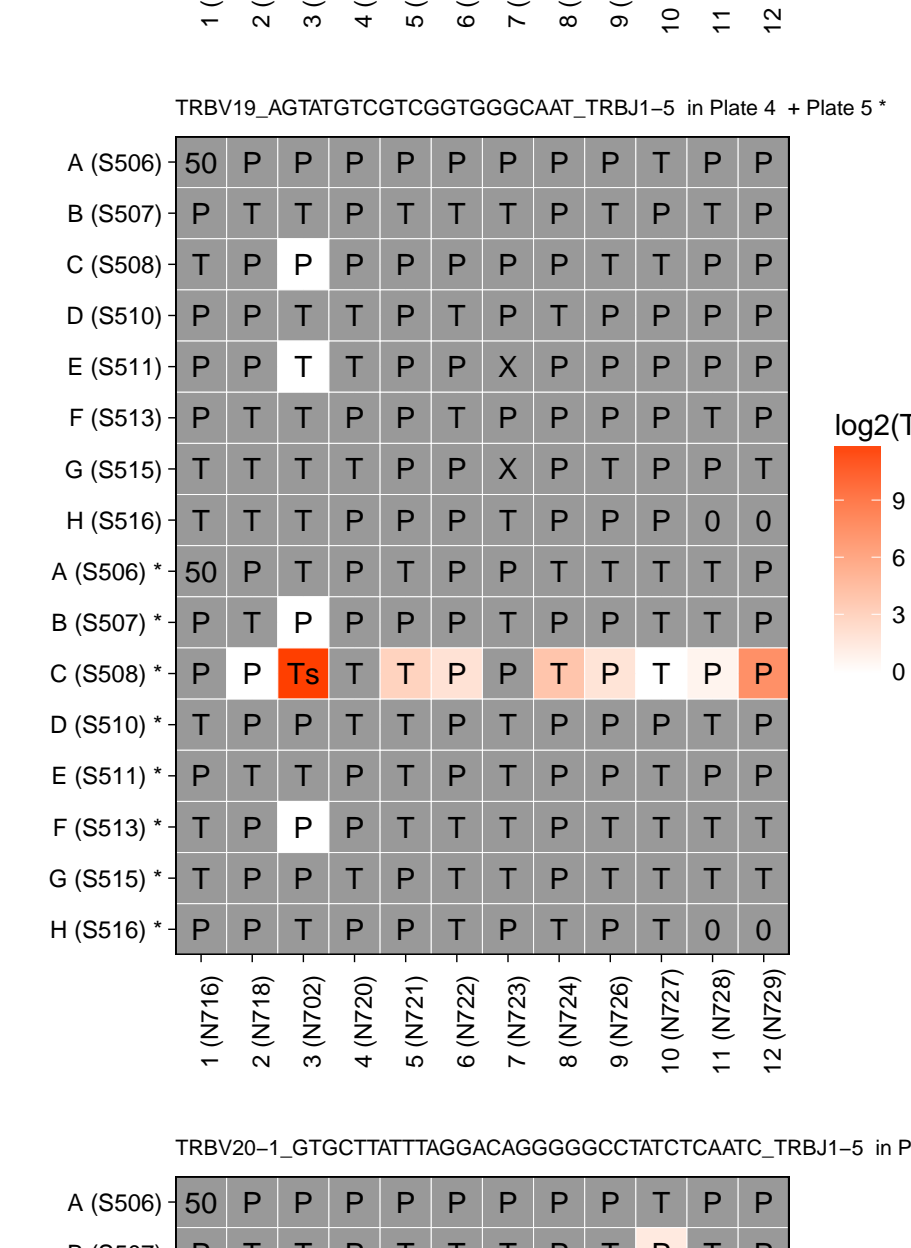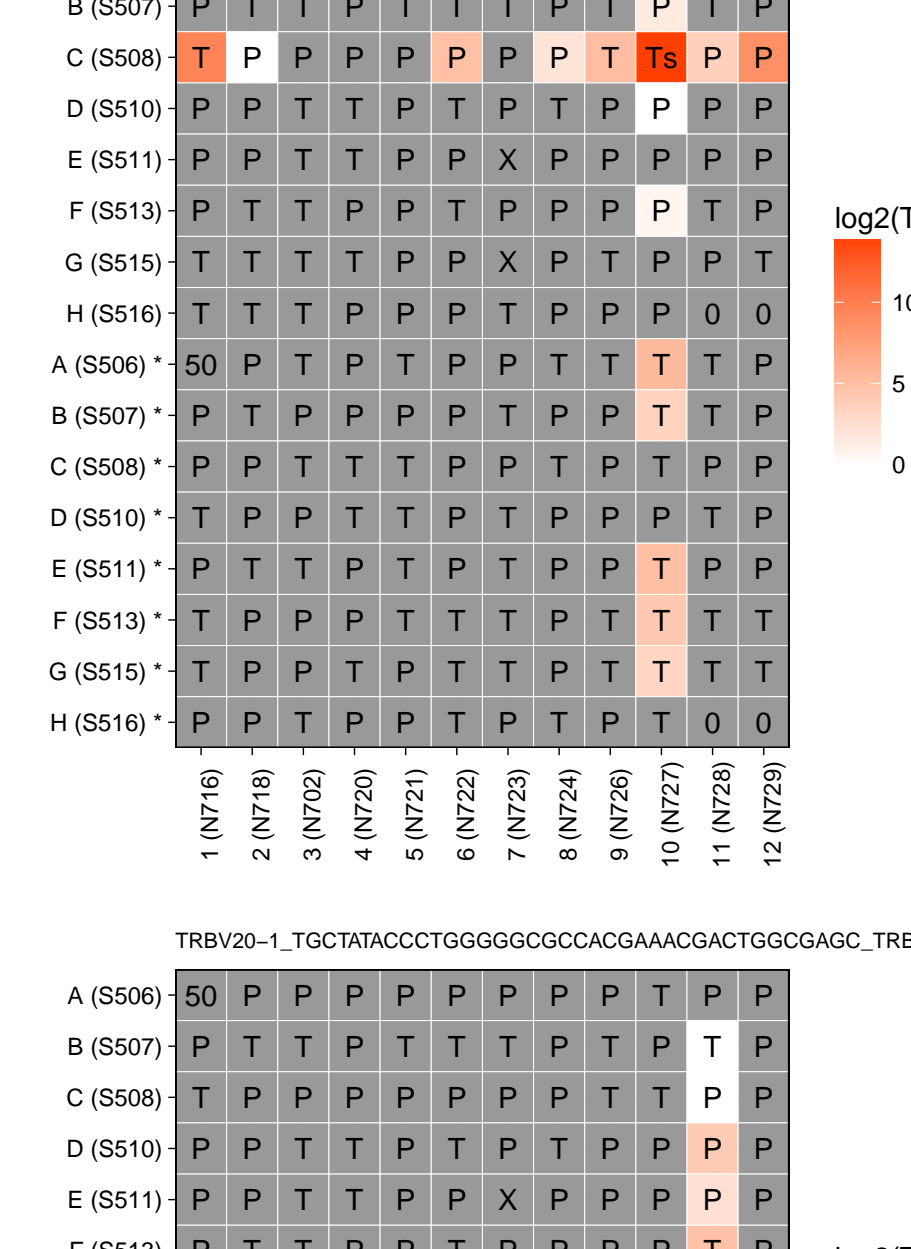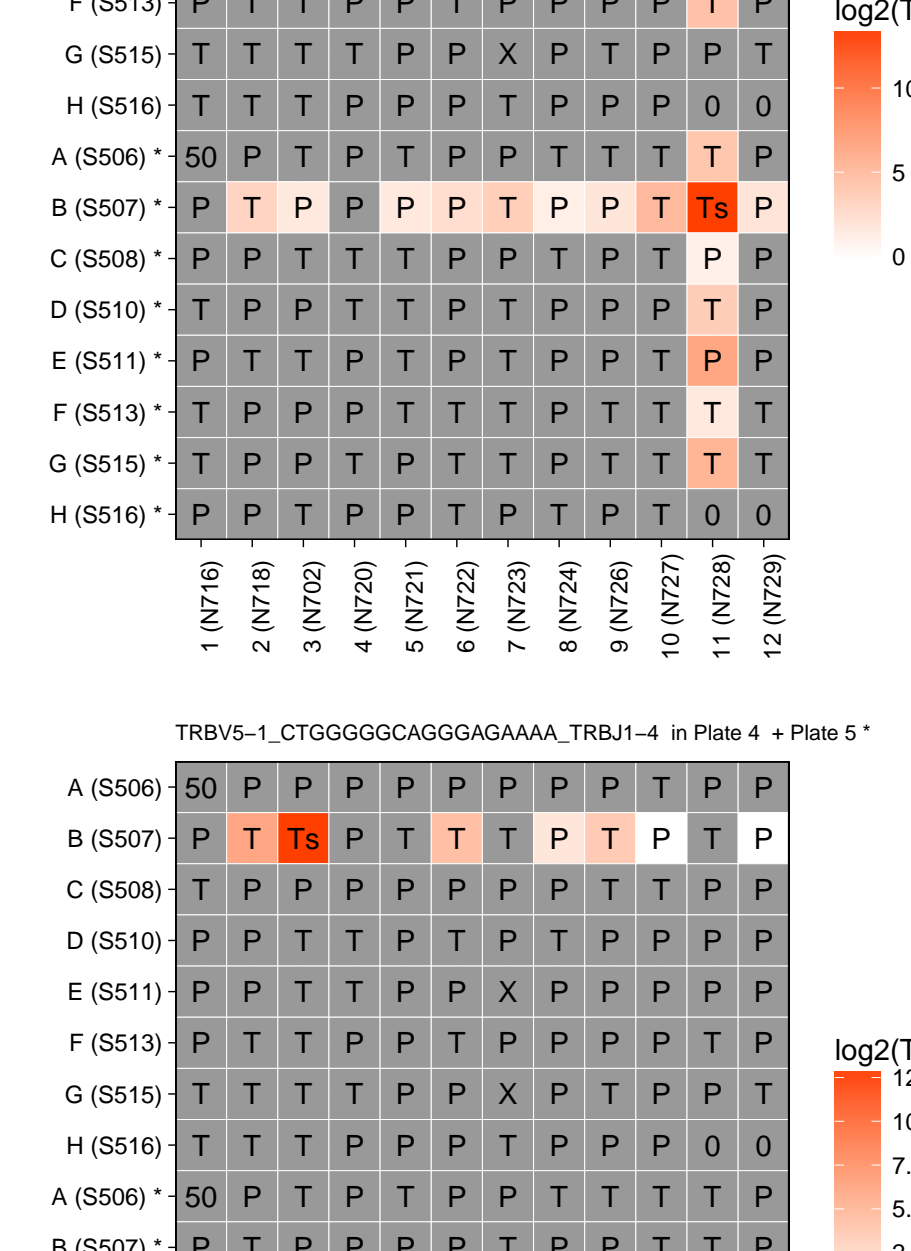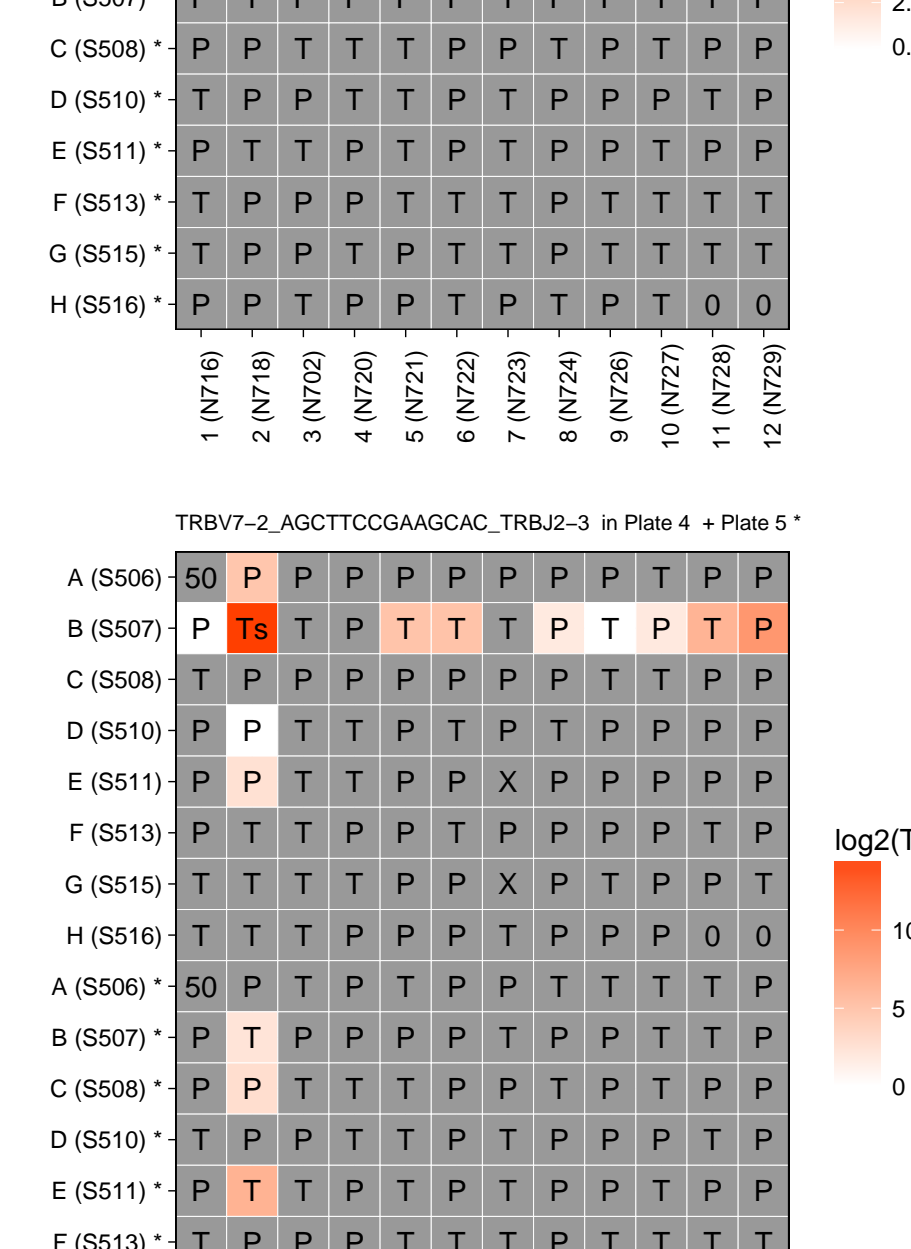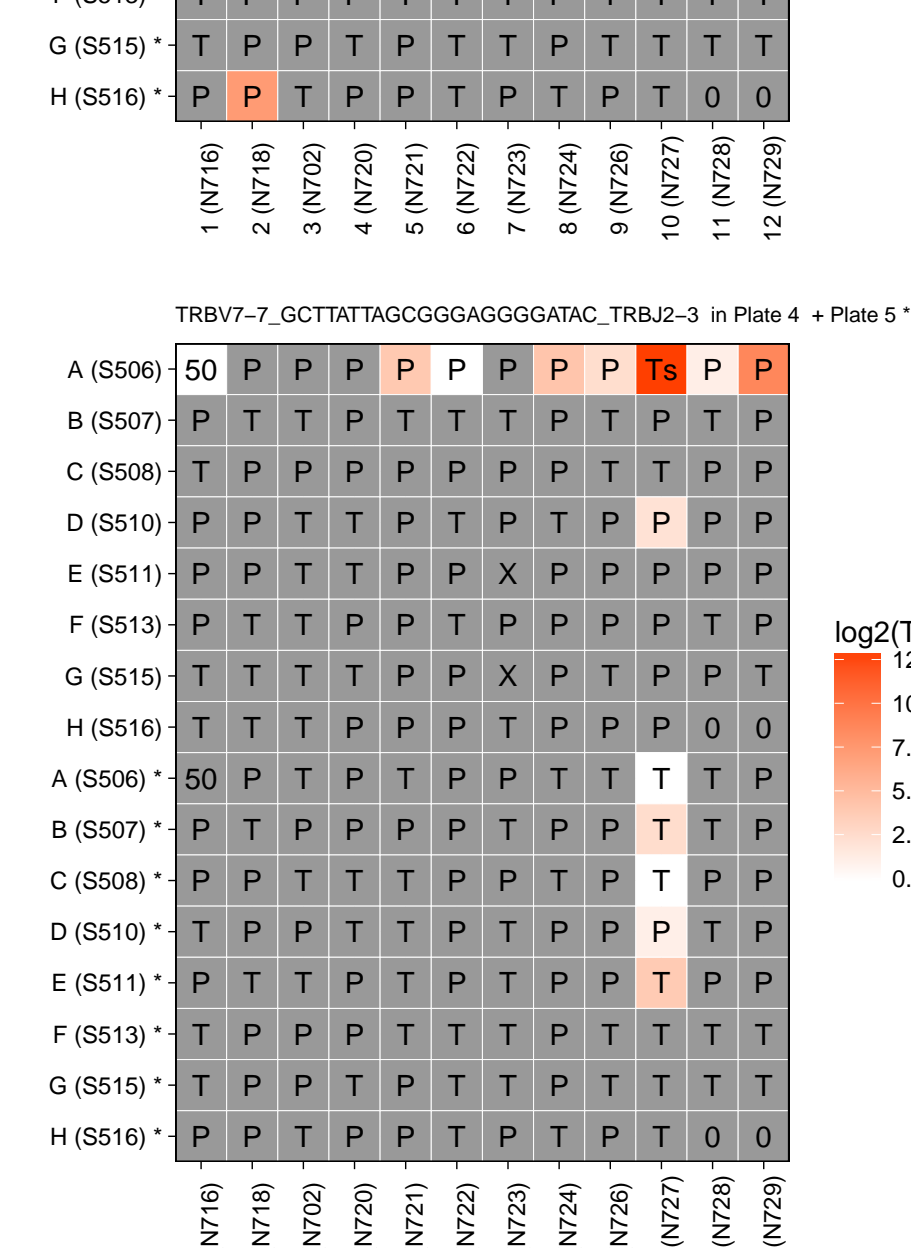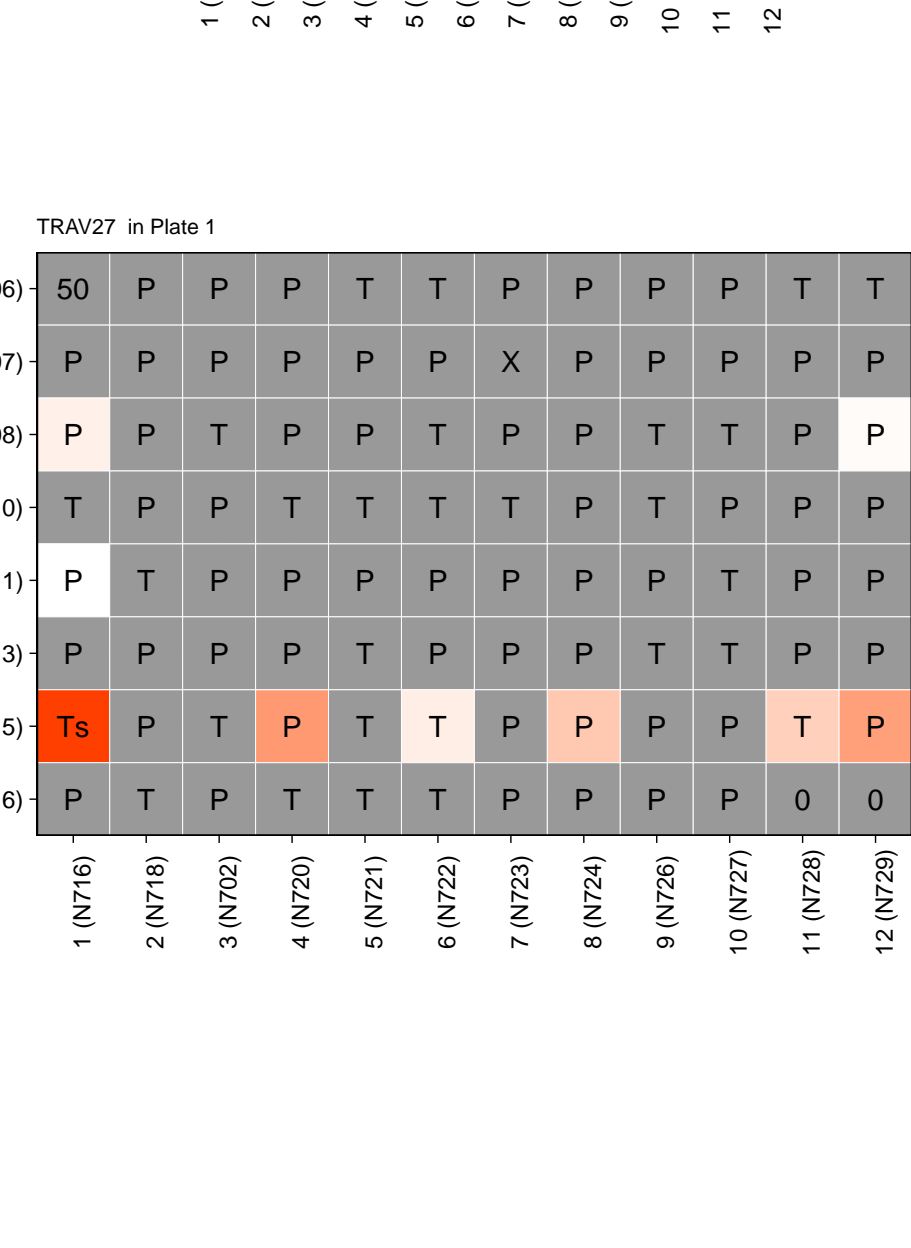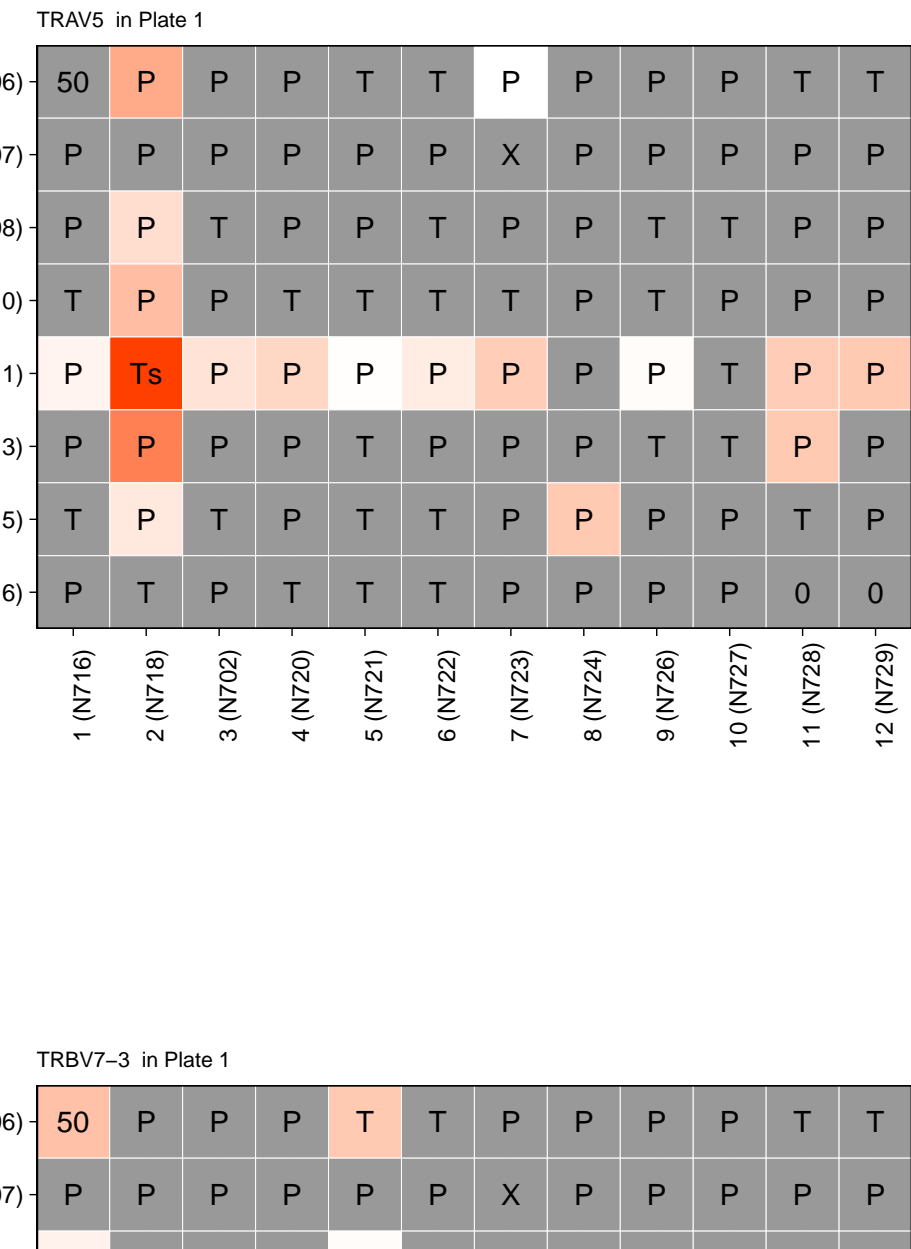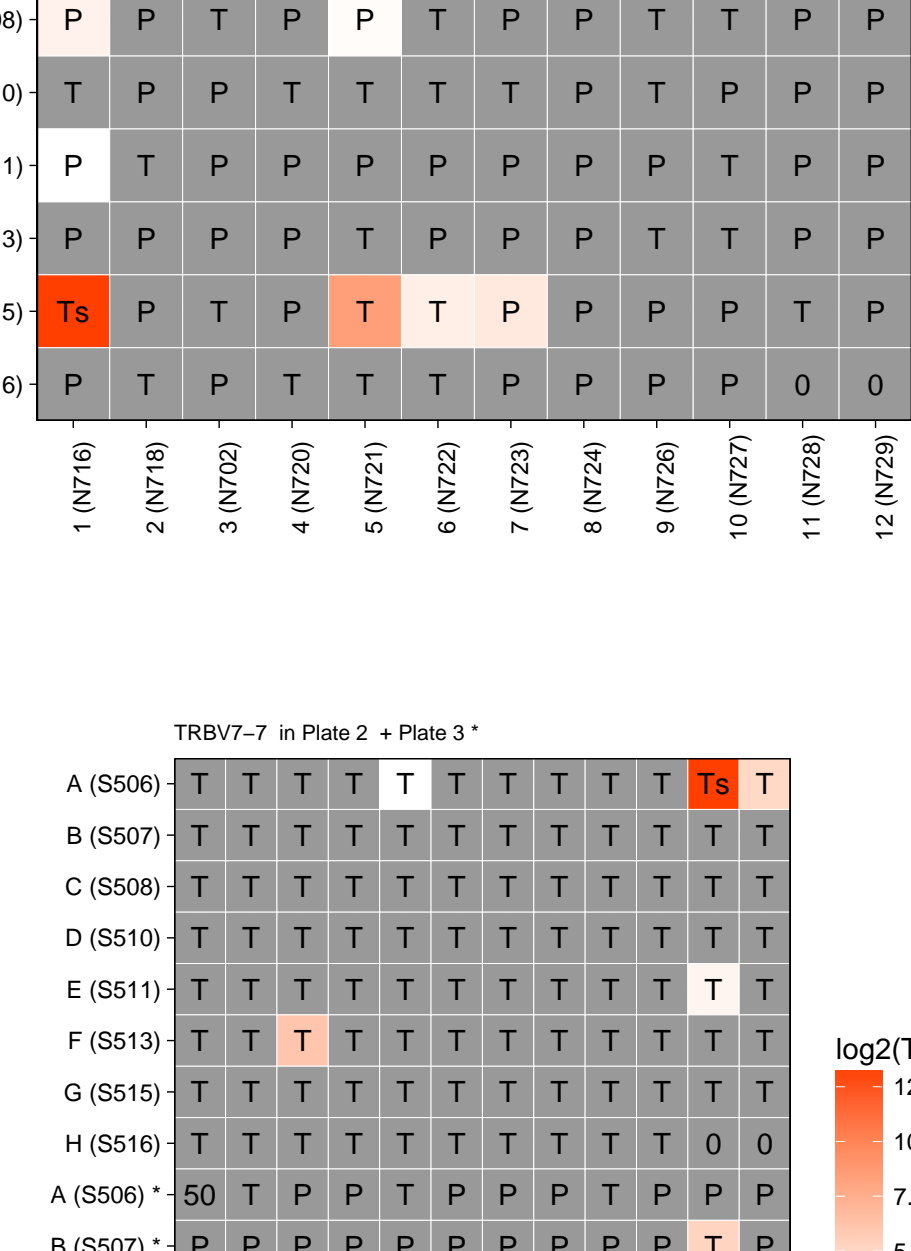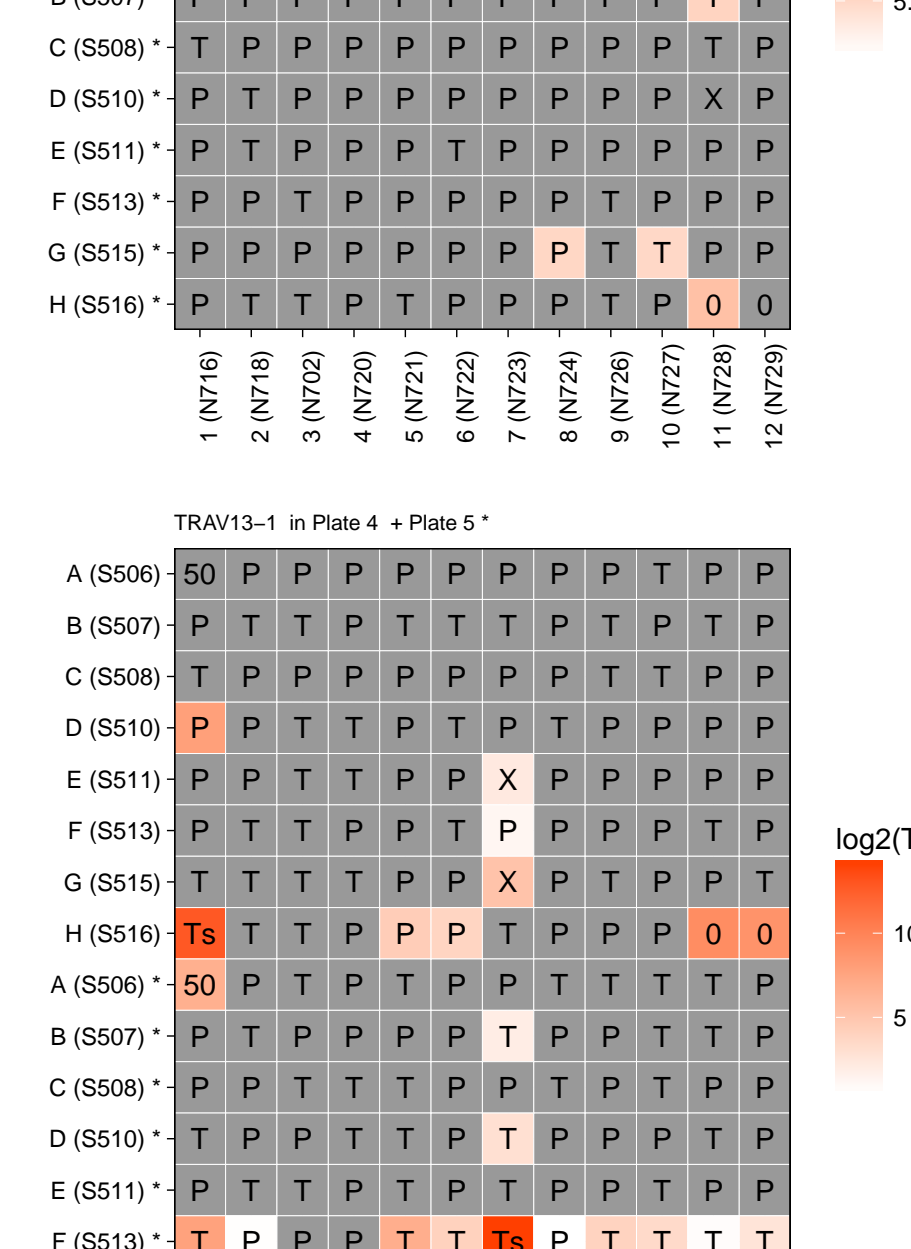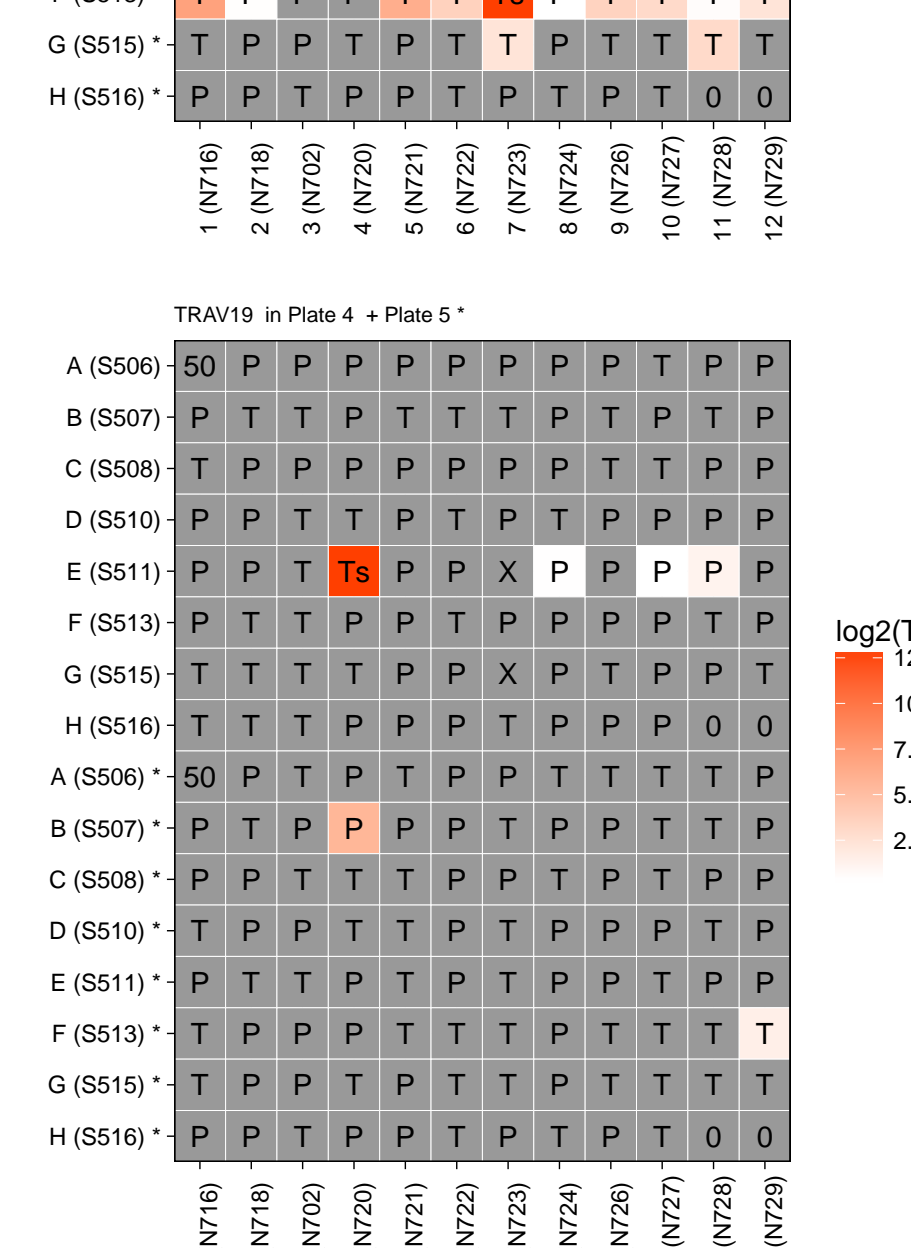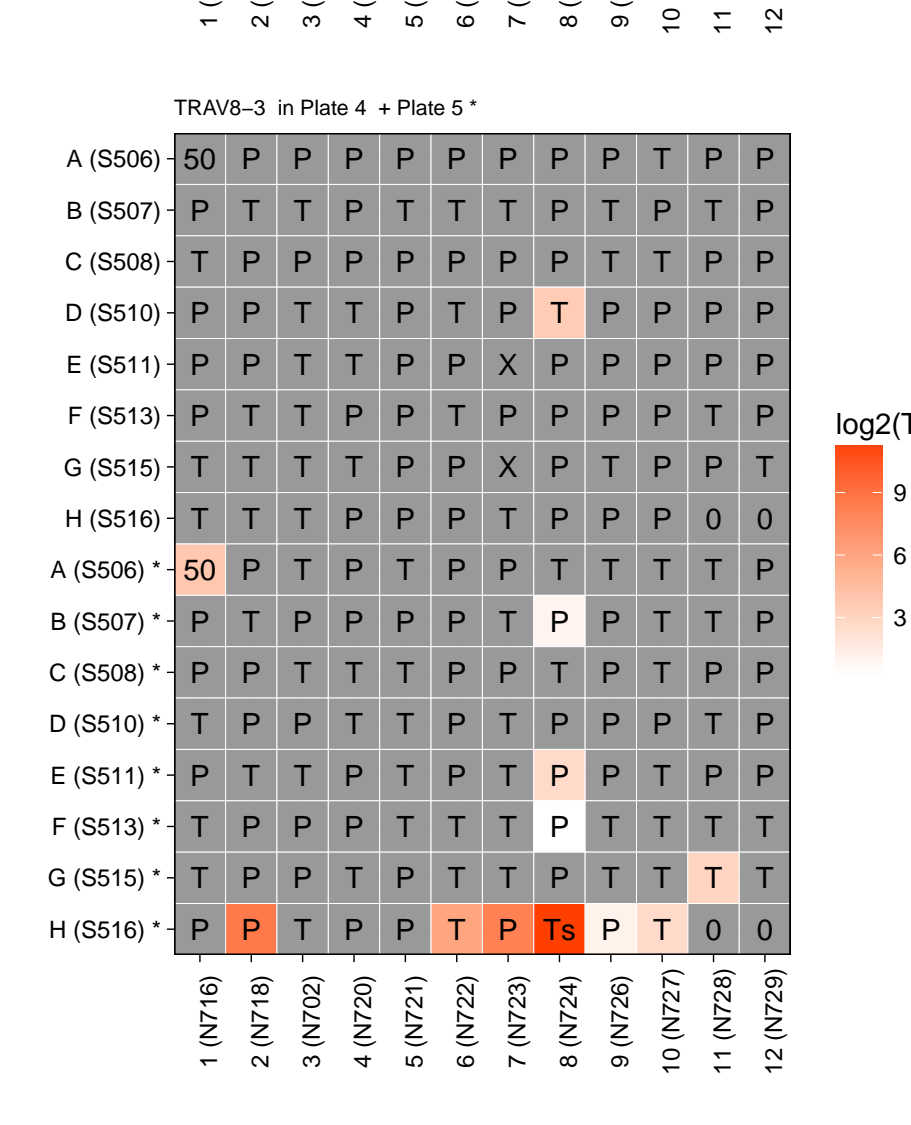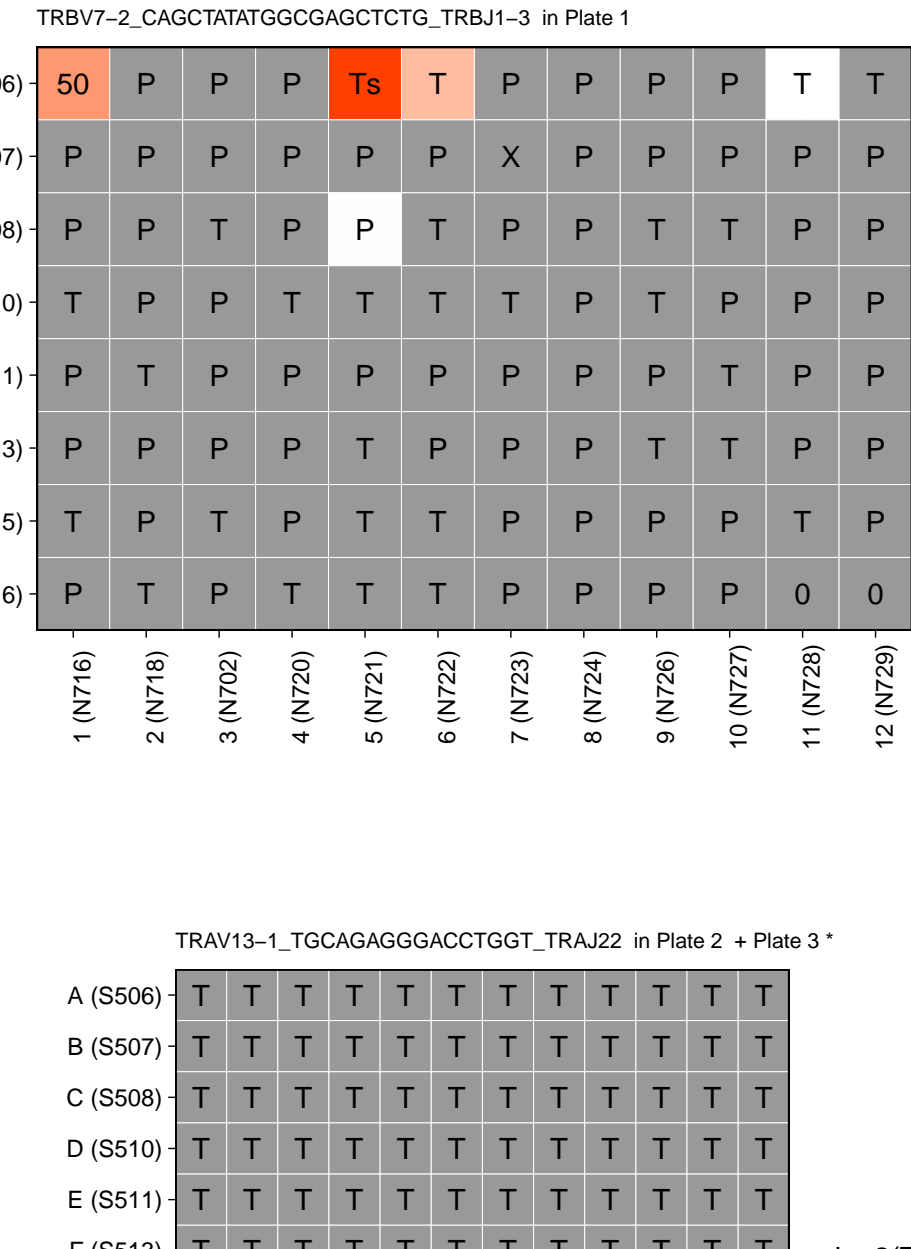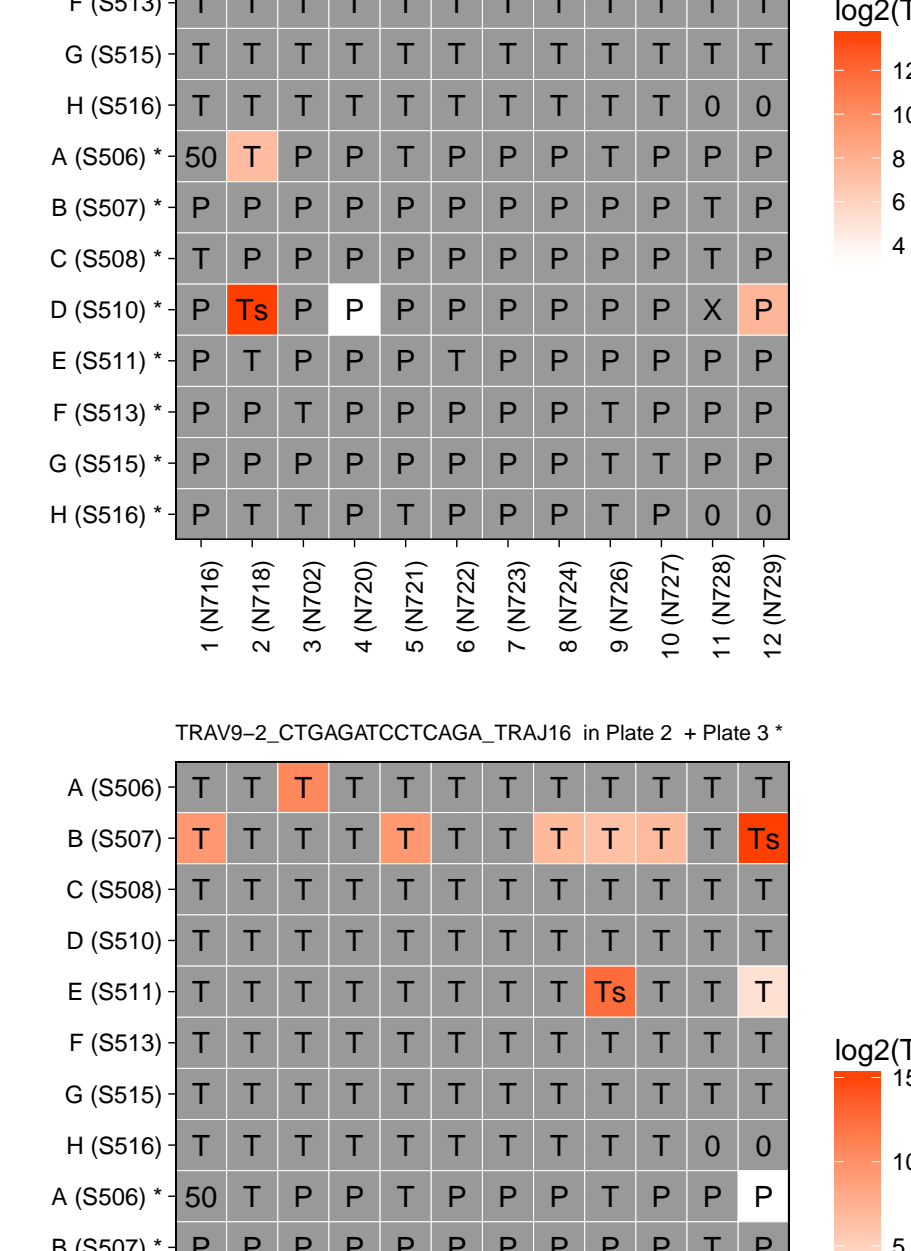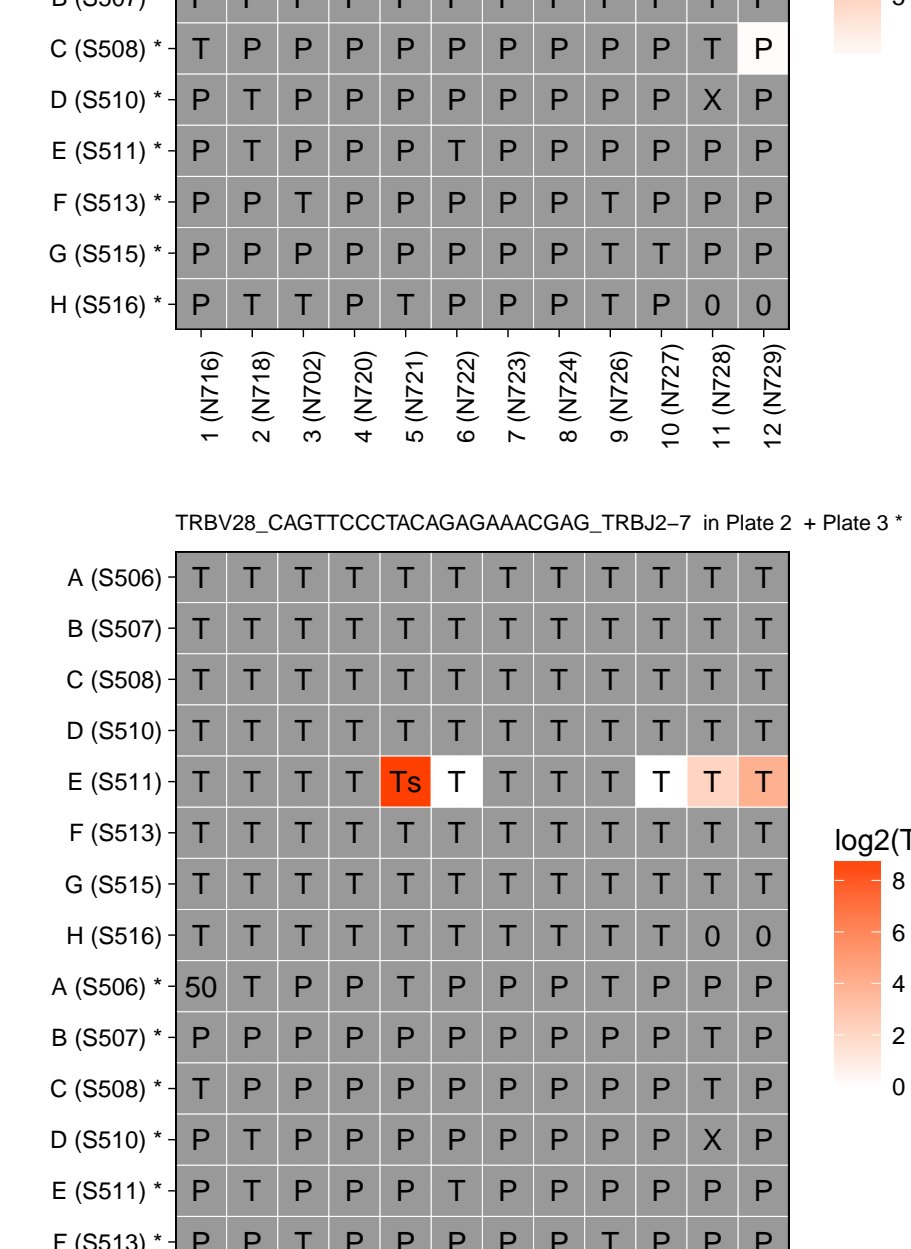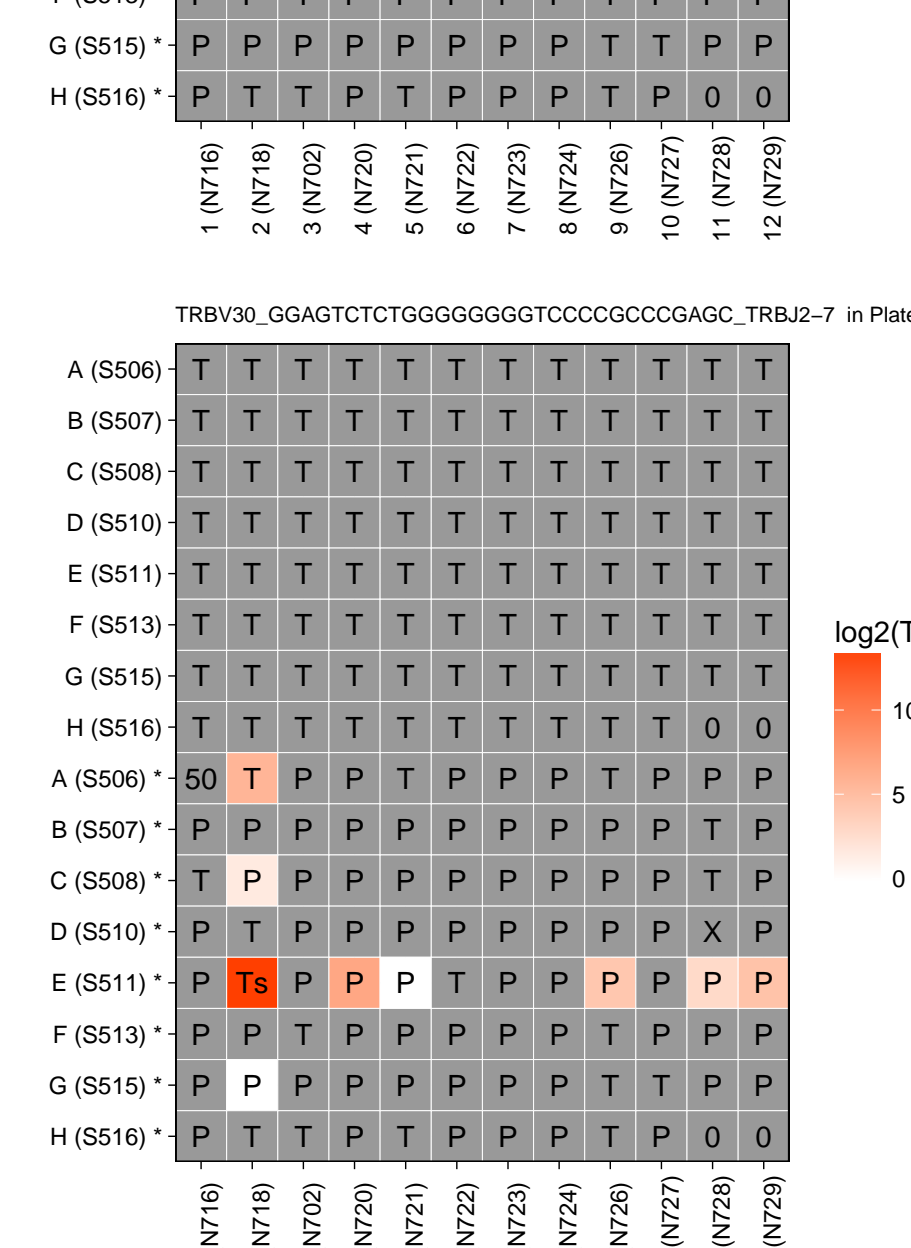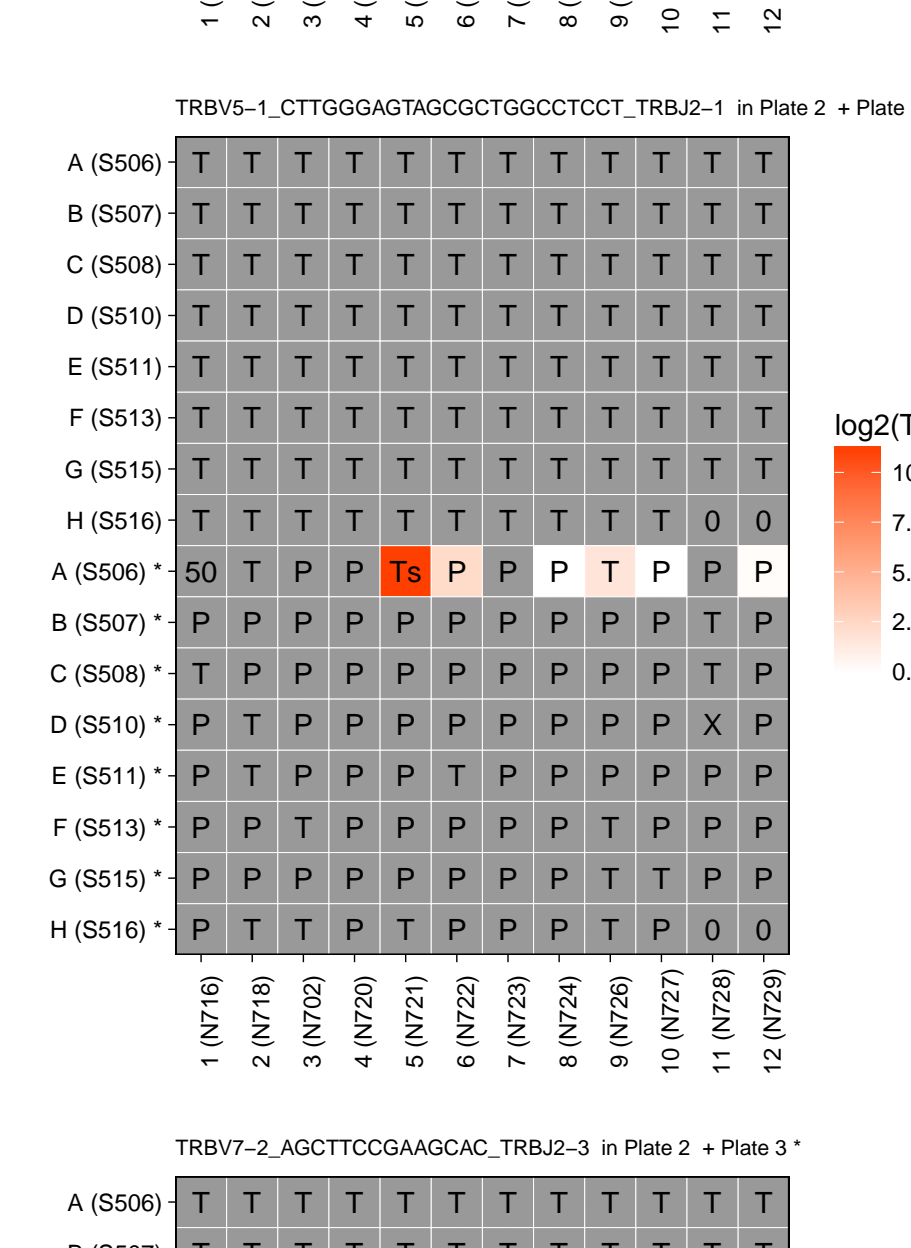

Supplement: S1 Fig — Cell type is labeled in the corresponding well, T for T cell, P for plasma cell, 0 for empty, 50 for mixture of multiple cells and X for unknown type. Ts for T cell identified as a source of signal spreading. (PDF) [file pone.0208484.s003.pdf]

Batch 1

Plate 1

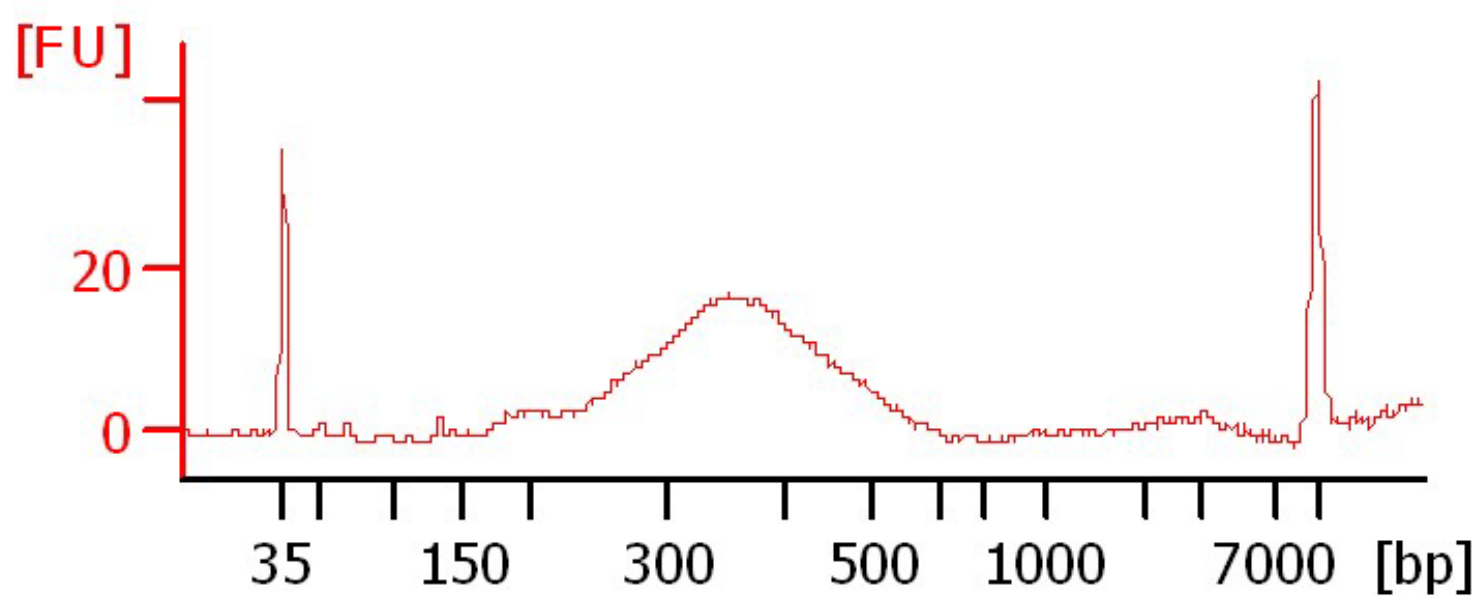

Batch 2

Plate 2

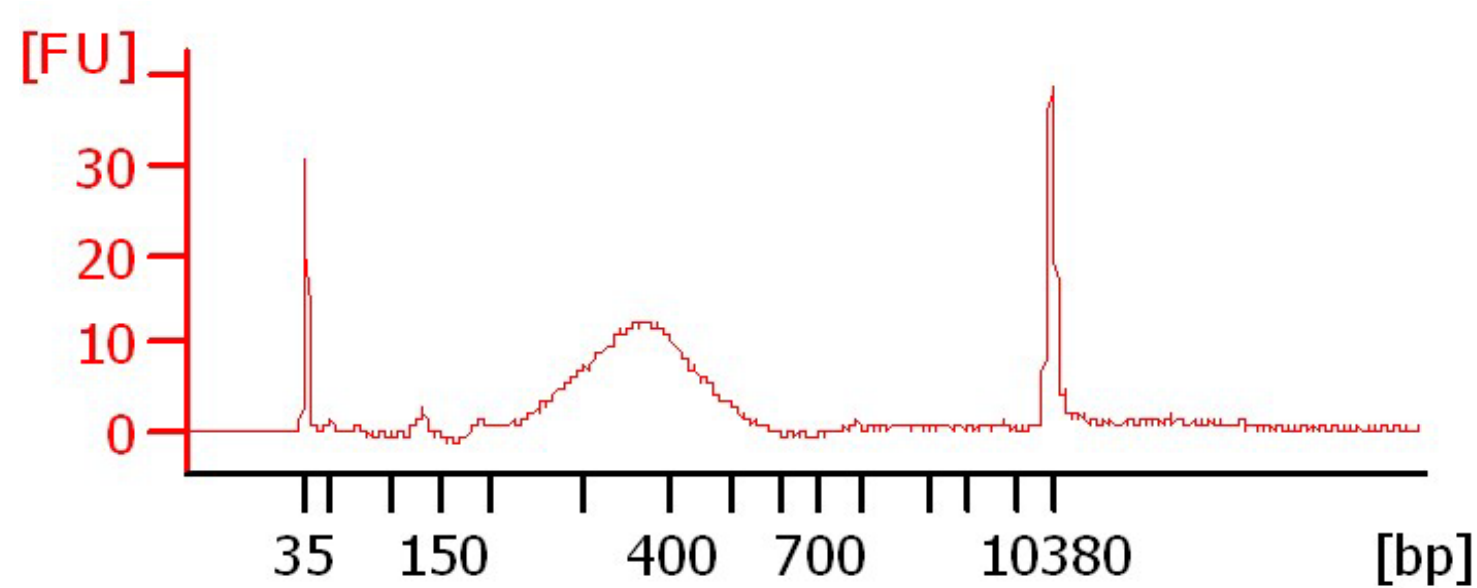

Batch 2

Plate 3

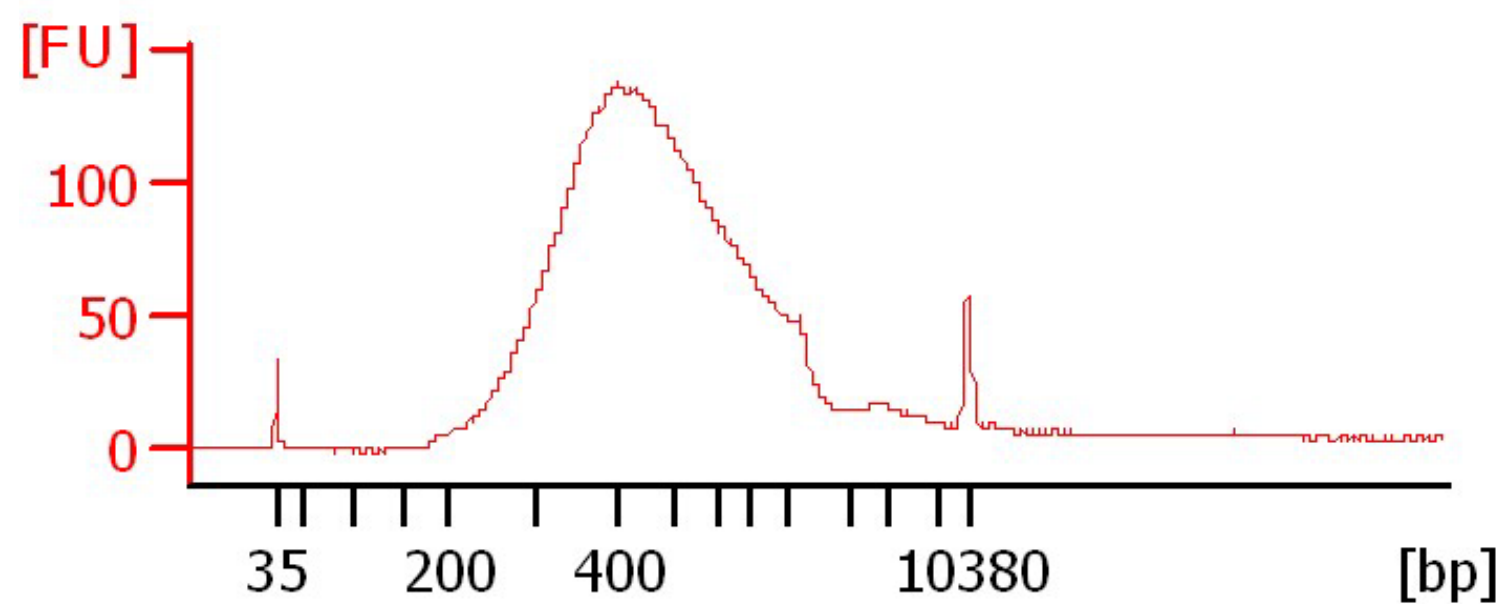

Batch 3

Plate 4

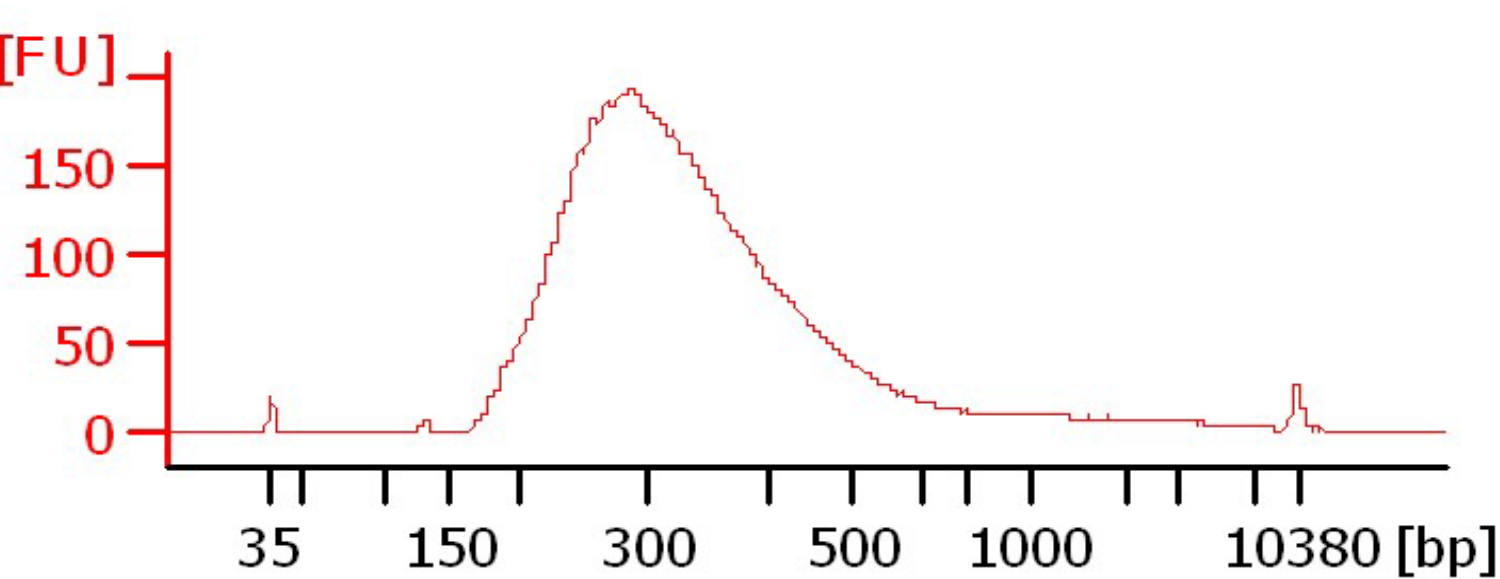

Batch 3

Plate 5

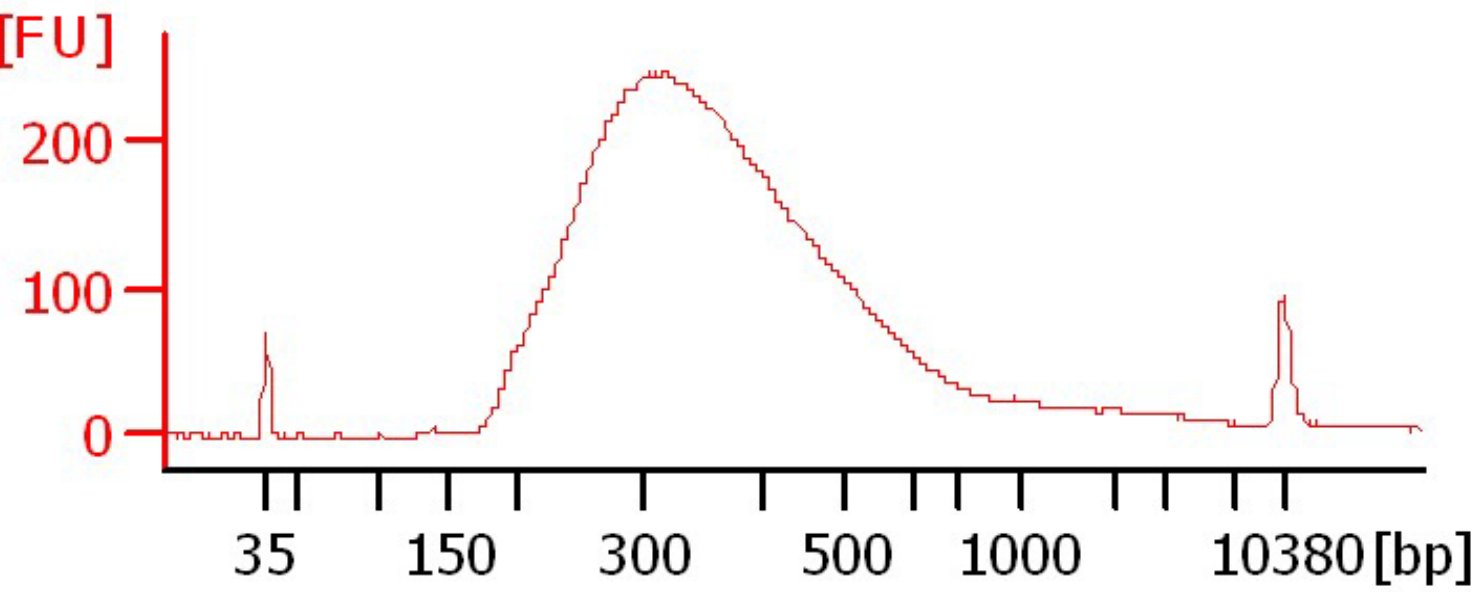

Supplement: S2 Fig — After Tagmentation, adaptors including indices were incorporated by PCR. The 96 samples from each plate were then pooled and free primers were removed by two rounds of purification with AMPure XP beads. The pooled libraries were quality-checked on the BioAnalyzer by the High-sensitivity DNA kit before kept frozen at -20C until sequencing. (PDF) [file pone.0208484.s004.pdf]
